# Fusobacterium mortiferum and its metabolite 5-aminovaleric acid promote the development of colorectal cancer in obese individuals through Wnt/β-catenin pathway by DKK2

**DOI:** 10.1080/19490976.2025.2502138

**Published:** 2025-05-08

**Authors:** Jiaxin Deng, Jiawei Zhang, Mingli Su, Juan Li, Yuping Su, Qinghua Zhong, Jiancong Hu, Yongcheng Chen, Sen Liao, Dezheng Lin, Xuefeng Guo

**Affiliations:** a Department of Endoscopic Surgery, The Sixth Affiliated Hospital, Sun Yat-sen University, Guangzhou, China; b Guangdong Provincial Key Laboratory of Colorectal and Pelvic Floor Diseases, The Sixth Affiliated Hospital, Sun Yat-sen University, Guangzhou, China; c Biomedical Innovation Center, The Sixth Affiliated Hospital, Sun Yat-sen University, Guangzhou, China

**Keywords:** Colorectal cancer, Fusobacterium mortiferum, obese individuals, DKK2

## Abstract

Colorectal cancer (CRC) is one of the most prevalent cancers worldwide, with high incidence and mortality rates. An increasing body of research suggests that obesity is a significant risk factor for the development of CRC. Moreover, recent findings have highlighted the close association between the gut microbiota and both obesity and CRC. Despite this, the specific mechanisms by which the gut microbiota influences obesity and CRC remain unclear. This study aims to explore the role of the gut bacterium Fusobacterium mortiferum and its metabolite 5-aminovaleric acid (5-AVA) in the development of obesity and CRC. Our study found that the metabolite 5-aminovaleric acid produced by Fusobacterium mortiferum significantly inhibits the expression of the tumor suppressor DKK2. This inhibition leads to enhanced proliferation of CRC cells. Furthermore, we discovered that Fusobacterium mortiferum and 5-AVA can activate the Wnt/β-catenin signaling pathway by inhibiting DKK2, thereby promoting tumor growth. This finding was validated in CRC mouse models and in vitro experiments. Additional mechanistic studies revealed that 5-AVA interacts with the demethylase KDM6B, affecting the demethylation process of DKK2 and subsequently activating the Wnt/β-catenin signaling pathway. Our study retrospectively collected fecal samples from patients who underwent gastrointestinal endoscopy at the Sixth Affiliated Hospital of Sun Yat-sen University over the past five years. Participants were stratified into a healthy control group and an adenoma group based on the outcomes of their colonoscopies. Following this, we conducted metagenomic analysis to identify differential bacteria, and based on the results, we performed bacterial cultivation and metabolomic profiling. The roles of the targeted bacteria and their metabolites were further validated through animal models and cellular assays, employing techniques such as Western Blot, qPCR, immunohistochemistry, molecular docking simulations, and gene overexpression studies. This study uncovers the potential carcinogenic effects of Fusobacterium mortiferum and 5-AVA in the development of obesity and CRC. Our research emphasizes the complex interplay between the gut microbiota and host metabolism and suggests new directions for future research to explore how modulation of the gut microbiota could prevent and treat CRC.

## 1. Introduction

Colorectal cancer is one of the most common malignant tumors of the digestive tract at present.^1,2^ The occurrence of colorectal cancer is related to various mechanisms, such as the Notch signaling pathway,^3^ immune escape,^4^ and ferroptosis,^5^ but the specific mechanisms of its occurrence are still unclear. Obesity is a chronic digestive system disease related to multiple systems,^6^ and a large amount of evidence suggests that obesity is one of the risk factors for the occurrence of colorectal cancer.^7^ Obesity is associated with a chronic, low-grade inflammatory state, which may also lead to the development of cancer.^8,9^

The human gut microbiome is an ecosystem composed of bacteria, fungi, viruses, archaea, and parasites.^10^ Human gut microbiota, as the main component of the gut microbiome, regulates the occurrence and development of colorectal cancer by releasing various metabolic products, proteins, and large molecules to interact with the host’s colonic epithelial and immune cells.^11^ The gut microbiota can serve as a noninvasive biomarker for predicting colorectal cancer; treatments based on the gut microbiota such as probiotics, fecal microbiota transplantation, and phage targeting of pathogenic bacteria also have clinical translation significance.^12^ Changes in the gut microbiota play an important role in diseases such as colorectal cancer^13^ and significantly affect obesity.^14^ Displacement of the gut microbiota, the formation of an inflammatory microenvironment, and secretion of microbiota-related metabolites may promote the occurrence of obesity, and further affect colorectal diseases, forming a vicious cycle.^15^

Fusobacterium mortiferum (Fm.) is a member of the Clostridium genus. Metagenomic results suggest that Fusobacterium may be related to the occurrence of colorectal cancer.^16^ However, there are currently no literature reports on the mechanism of Fusobacterium mortiferum in the occurrence of colorectal cancer in obese populations. Previous literature reports that populations enriched with Fusobacterium mortiferum have increased levels of some human metabolites in serum. Among them, 3-indolepropionic acid has the potential to protect primary neurons and neuroblastoma cells from oxidative damage and death, but its role in the colon has not been studied^17^; d-aminobutyric acid may be involved in the “adenoma-carcinoma sequence” paradigm,^18^ and the recognized “adenoma-carcinoma sequence” plays an important role in the development of colorectal cancer^19^; 2-deoxy-d-ribose is involved in the development of human tumors by promoting angiogenesis,^20^ and is significantly positively correlated with Fusobacterium mortiferum. Fusobacterium mortiferum may be closely related to APC mutations,^21^ the mutation of the APC gene is usually associated with the activation of the Wnt/β-catenin pathway,^22^ but the specific mechanism remains unclear.

Dickkopf 2 (DKK2), recognized for its secretion and characterized by two domains rich in cysteine separated by a connecting segment,^23^ is part of the Dickkopf family and has been demonstrated to play a pivotal role in modulating Wnt/β-catenin signaling pathways.^24^ In the landscape of cancer research, DKK2 has emerged as a novel oncogenic factor implicated in a spectrum of malignancies,^25,26^ notably in colorectal cancer where it is implicated in enhancing cell proliferation and invasiveness via the Wnt/β-catenin signaling mechanism.^23^ In the context of colorectal cancer, DKK2 is frequently detected in a hypermethylated state within tumor tissues and cellular specimens, a condition that correlates with reduced mRNA expression levels and a substantial influence on tumorigenesis.^27^ Moreover, DKK2 exerts its influence beyond cancer biology, functioning as an adipokine that curbs the synthesis of fat and significantly contributes to the pathogenesis of obesity.^28,29^ Adipose tissue, in addition to its energy storage role, is capable of secreting a range of bioactive substances and adipokines with pro-inflammatory properties.^30^ These secreted factors have the potential to perpetuate a state of chronic low-grade inflammation within the intestinal tract. The interplay of obesity, gut microbiota dysbiosis, and alterations in intestinal permeability may exacerbate this inflammatory state,^31^ and sustained inflammatory pressures coupled with shifts in the microbial composition are posited to potentially precipitate tumorigenesis,^32^ thereby establishing a self-reinforcing cycle of pathological change. In the realm of epigenetics, KDM6B stands out as a histone demethylase that is integral to the demethylation of H3K27me3.^33^ Its dysregulated expression is observed across various types of cancer, with varying implications for the biological behavior of tumors and their clinical outcomes.^34–36^ The diverse manifestations of KDM6B’s role underscore the complexity of its influence in the cancerous process.^37^

This study is based on the significant difference in the abundance of Fusobacterium mortiferum between obese adenoma patients and obese non-adenoma populations, aiming to explore whether Fusobacterium mortiferum and its metabolic product 5-aminovaleric acid can promote the occurrence of colorectal cancer in obese populations by inhibiting KDM6B-mediated DKK2 demethylation and activating the Wnt/β-catenin pathway.

### 2. Materials and methods

The Definitions of Obesity: Obesity was defined as a Body Mass Index (BMI) of 25 kg/m^2^ or higher, according to the World Health Organization (WHO) criteria for Asian populations.^38,39^

#### 2.1. Sample collection

Microbial community analysis samples were collected through the fecal matter of 40 obese individuals and 40 non-obese individuals. In subsequent analyses, it was found that among the obese population, 20 had adenomas, while 20 obese individuals showed no adenomas in their intestines. The diagnosis of adenomas was based on an integrated assessment of endoscopic observations, clinical data, and histopathological findings. The clinical information of the recruited cohort is listed in Table S1 of the Supporting Information. The diagnosis of colorectal cancer was made according to clinical guidelines and typical histopathological results. Exclusion criteria included patients who were unwilling to provide informed consent, those who had taken antibiotics within two weeks, patients who were contraindicated for endoscopic examination, those with acute gastrointestinal infections, pregnant patients, patients with known bleeding disorders, or those with a history of gastrointestinal surgery. Fecal samples were collected strictly following the established procedures, immediately transferred to centrifuge tubes after collection, and stored at − 80°C. The samples were transported to the central laboratory of the authors’ hospital on ice within 30 minutes. The research protocol was approved by the Ethics Committee of the Sixth Affiliated Hospital of Sun Yat-sen University in Guangzhou, China (No.2021ZSLYEC–206).

### 2.2. Metagenomic sequencing and analysis

We retrieved our internal metagenomic data to examine the abundance of Fusobacterium mortiferum in human fecal samples. Genomic DNA from human feces was extracted using the PowerSoil Pro Kit (Qiagen, Hilden, Germany), followed by shotgun metagenomic sequencing on the Illumina Novaseq platform, performed by WEKEMO Company in Shenzhen, China. Raw reads were inspected using KneadData to ensure the data consisted of high-quality, contamination-free microbial reads. The number of sequences of species present in the samples was calculated using Kraken2 in conjunction with our proprietary microbial nucleic acid database, while the actual abundance of species in the samples was estimated using Bracken. In this study, LEfSe analysis was performed with rigorous statistical correction. Specifically, normalization scaling factors were applied, which serves as a data preprocessing technique to correct for variations in sequencing depth or technical biases among samples. Furthermore, the LDA Score threshold and the Kruskal-Wallis statistical method were utilized to ensure the reliability of the results. In the heatmaps of the grouped clustering, the z-score normalization method was employed. This method standardizes the data by transforming it to have a mean of zero and a standard deviation of one, thereby enabling comparability across different features or samples.

### 2.3. Non-targeted metabolite analysis

The analysis was conducted by WEKEMO Company, located in Shenzhen, China. The metabolite profiling was performed utilizing a Dionex Ultimate 3,000 RS UHPLC system, integrated with a Q Exactive quadrupole Orbitrap mass spectrometer and a heated electrospray ionization (ESI) source, all from Thermo Fisher Scientific, based in Waltham, MA, United States. This setup facilitated the analysis of metabolic profiles in both positive and negative ESI modes. An ACQUITY UPLC HSS T3 column (1.8 μm, 2.1 mm × 100 mm, Waters, United Kingdom) was utilized for chromatographic separation in dual polarity modes. The mobile phase was a binary gradient system comprising (A) water with 0.1% formic acid by volume (v/v) and (B) acetonitrile with 0.1% formic acid by volume (vol/vol). The system started with an initial solvent composition of 95% A and 5% B. The gradient elution was programmed as follows: holding 5% B from 0.01 to 2 minutes, increasing to 30% B by 4 minutes, further to 50% B by 8 minutes, then to 80% B by 10 minutes, and finally to 100% B by 14 minutes. The system maintained 100% B for an additional 2 minutes, followed by a reequilibration step back to the initial conditions (100–5% B) over 15 to 15.1 minutes, and a stabilization period at 5% B from 15.1 to 16 minutes. Quality control (QC) samples were injected at consistent intervals after every 10 samples throughout the sequence to ensure analytical consistency and repeatability. The resultant LC-MS raw data were processed using progenesis QI software from Non-linear Dynamics, based in Newcastle, United Kingdom. Metabolite identification was accomplished with the aid of progenesis QI Data Processing Software by Waters Corporation, Milford, United States. Differential metabolites were screened using a Random Forest model combined with a t-test (*p* < 0.05).

### 2.4. Bacterial cultivation protocol

The strains Fusobacterium mortiferum (ATCC25557) and Escherichia coli (MG1655) were procured from the American Type Culture Collection. Cultivation of Fusobacterium mortiferum was conducted under anaerobic conditions at a temperature of 37°C for a duration of 24 hours, utilizing ATCC medium 2107. Post cultivation, the bacterial cells were harvested and resuspended in brain-heart infusion (BHI) broth. Concurrently, E. Coli MG1655 was grown under aerobic conditions at the same temperature in BHI broth, with an incubation period of 24 hours.

### 2.5. Animal experiments

For the experimental procedure, male C57BL/6 mice at six weeks of age were chosen. The mice received weekly intraperitoneal injections of azoxymethane (AOM) at a dosage of 10 mg/kg for a period of six weeks. Commencing two weeks prior to the initiation of gavage, the drinking water provided to the mice was enriched with a cocktail of antibiotics, comprising penicillin, streptomycin, and metronidazole at a concentration of 0.2 g/L each, along with vancomycin at 0.1 g/L. Subsequent to the AOM injections, a daily gavage of 200 μL of Fusobacterium mortiferum, standardized to 1 × 10^8^ colony-forming units (CFU), was administered to the mice over a 20-week period to elicit tumorigenesis. A concurrent bacterial control group was given an equivalent volume of E. Coli MG1655 via the gavage route, whereas a blank control group was treated with 200 μL of brain-heart infusion medium devoid of Fusobacterium mortiferum. At the culmination of the 20-week experimental period, the mice were humanely euthanized to evaluate the effects of bacterial induction on tumor formation. All animal experiments were conducted following the guidelines and were approved by the Ethics Committee of HuaTeng Company (Approval Number: C202301–3).

### 2.6. Cell cultivation

The colorectal adenocarcinoma cell lines HCT116 and DLD1 were procured from the American Type Culture Collection (ATCC). These cell lines were maintained in Dulbecco’s Modified Eagle Medium (DMEM), which was obtained from Gibco BRL, Grand Island, NY, and enriched with 10% Fetal Bovine Serum (FBS) sourced from Thermo Fisher Scientific, Waltham, MA, as well as 1% penicillin/streptomycin to ensure sterility. The cells were cultivated under controlled environmental conditions at 37°C within a humidified incubator that facilitated a 5% CO2 atmosphere.

### 2.7. Bacterial cell co-culture

Human colorectal cancer cells HCT116 and DLD1 were co-cultured with bacteria (Fusobacterium mortiferum or E.coli) under conditions of 37°C and 5% CO2, at a multiplicity of infection (MOI) of 10, for a duration of 4 hours. Subsequently, the cells were washed with warm PBS containing antibiotics (kanamycin and ampicillin at a concentration of 100 ng/mL each). The cells were then further cultured for an additional hour in DMEM (Gibco) supplemented with the aforementioned antibiotics. Following this, assays for cell proliferation and clonal formation were conducted. RNA from the cells was extracted using TRIzol reagent (Thermo Fisher Scientific), and gene expression was measured by quantitative PCR (qPCR).

### 2.8. 5-AVA co-culture with cancer cells

Human colorectal cancer cells HCT116 and DLD1 were co-cultured with 5-AVA or PBS (at a concentration of 0.1 mg/L) under conditions of 37°C and 5% CO2 for a period of 12 hours. Following the co-culture, the cells were washed with warm PBS. Subsequently, assays for cell proliferation and clonal formation were carried out. RNA was extracted from the cells using TRIzol reagent (Thermo Fisher Scientific), and gene expression levels were measured by qPCR.

### 2.9. Assessment of cell proliferation and clonogenicity

Colorectal carcinoma cells were seeded into a 96-well plate at a concentration of 2000 cells per well for the proliferation assay and incubated for an initial period. The IncuCyte Zoom system was employed for time-lapse imaging every 24 hours, and proliferation was quantified by the confluence metric derived from phase-contrast microscopy, with data analysis performed using IncuCyte’s analytical software. In the clonogenic assay, 400 colorectal carcinoma cells were seeded into each well of a 6-well plate and incubated to facilitate adhesion. After a culturing period of 1–2 weeks, the cells were fixed with paraformaldehyde for 30 minutes. Crystal violet staining was then applied for 30 minutes post-fixation. The staining solution was carefully removed with running water, and the cells were left to air-dry prior to image analysis using Image J software for quantification of clonal formation.

### 2.10. Transwell assay

The Transwell migration assay was conducted utilizing the Corning Transwell apparatus. HCT116 or DLD1 cells were initially seeded into the upper compartment of the Transwell insert in a 24-well format, with 100 μL of serum-free medium per well at a density of 1 × 10^6^ cells per well. The lower chamber was filled with 600 μL of medium supplemented with 30% FBS to serve as a chemoattractant. Subsequently, either PBS or a specified concentration of 5-AVA was introduced into the lower chamber. Following a 24-hour incubation period at 37 °C, the cells that had migrated to the lower surface of the membrane were fixed with a solution of formaldehyde and glacial acetic acid for 15 minutes. After fixation, the cells were rinsed with PBS and then stained with 0.1% crystal violet. The stained cells were visualized using a light microscope (10× magnification; Olympus Corporation, Tokyo, Japan), and the migration of HCT116 or DLD1 cells was quantified using the Image-Pro Plus 6.0 software.

### 2.11. Wound-healing assay

The migratory capacity of HCT116 cells was evaluated through a scratch wound-healing assay. Initially, the cells were seeded in 6-well plates and allowed to reach full confluence. A standardized scratch was introduced using a 200-microliter pipette tip to create a uniform wound. Subsequently, the cells were exposed to 5-AVA or PBS, in accordance with the experimental protocol, for a duration of 24 hours. The progression of wound closure was documented by acquiring images under a microscope at 100-fold magnification (Olympus Corporation, Tokyo, Japan). The relative healing of the wounded area was quantified by employing ImageJ software to determine the percentage of wound closure.

### 2.12. RNA sequencing and analytical approach

RNA was isolated from colorectal tissue samples, followed by the synthesis of complementary DNA (cDNA) and subsequent processes of adapter ligation and sample enrichment. Utilizing the Illumina Novaseq™ 6000 platform provided by LC-Bio Technology CO., Ltd. (Hangzhou, China), the RNA sequences were generated through a 2 × 150bp paired-end sequencing protocol as per the manufacturer’s guidelines. Post-sequencing, gene set enrichment analysis (GSEA) was conducted employing version 4.1.0 of the GSEA software in conjunction with the Molecular Signatures Database (MSigDB). This analysis aimed to discern significant gene expression disparities across defined gene ontology (GO) categories, KEGG metabolic and signaling pathways, Disease Ontology (DO) terms specific to Homo sapiens, and Reactome pathways relevant to select model organisms. The gene expression data matrix served as the foundational input for the GSEA, where genes were hierarchically ranked based on the Signal2Noise ratio, a normalization approach. The software’s standard parameters were applied to compute enrichment scores and corresponding p-values. GO categories, KEGG pathways, and associated Reactome pathways with an absolute normalized enrichment score (|NES|) exceeding 1, a nominal p-value (NOM p-val) less than 0.05, and a false discovery rate-adjusted q-value (FDR q-val) below 0.25 were deemed to exhibit significant differences between comparative groups.

### 2.13. Western blot

Total protein was extracted from tissue samples, and protein concentrations were determined using a MULTISKAN Sky spectrophotometer at an optical density wavelength of 562 nm. Subsequently, proteins were resolved via sodium dodecyl sulfate-polyacrylamide gel electrophoresis (SDS-PAGE) with gradients ranging from 8% to 12.5%. The resolved proteins were electrophoretically transferred onto a polyvinylidene fluoride (PVDF) membrane. The PVDF membrane was pre-treated with a 5% solution of nonfat milk in Tris-buffered saline with Tween-20 (TBST) at ambient temperature for 60 minutes to prevent nonspecific binding. Following this blocking step, the membrane was incubated with the primary antibody diluted to an appropriate concentration, under refrigeration at 4°C, for a duration of 16 to 24 hours to allow specific antigen-antibody interactions. After three rounds of washing with TBST to remove unbound primary antibodies, the membrane was incubated with a species-specific secondary antibody at room temperature for 60 minutes. Post-incubation, the membrane underwent another three washes with TBST to eliminate any nonspecifically bound secondary antibodies. Finally, the membrane was subjected to detection using a chemiluminescent gel imaging system, where it was exposed and developed in a darkroom environment to capture the immunoreactive bands.

### 2.14. Plasmid construction, siRNA, and transfection

HCT116 cells were transfected with a plasmid encoding the full-length human KDM6B gene (Addgene #24167) to achieve overexpression. Transfections were performed using LipoFiter™ reagent, following the manufacturer’s instructions. Cells were plated in 6 cm dishes, grown to 70–80% confluence, and then transfected with 5 μg of the KDM6B plasmid. The medium was refreshed 6 hours post-transfection, and cells were incubated at 37°C for 48 hours. Overexpression was validated by Western blotting and qPCR. siRNAs targeting KDM6B were synthesized by Guangzhou Riboio Co., Ltd. and transfected into cells using riboFECT™CP reagent according to the manufacturer’s protocol. Cells were seeded in six-well plates and grown for 24 hours before transfection. At 50% confluence, siRNAs were diluted in riboFECT™ CP buffer and mixed with the transfection reagent, then incubated for 15 minutes before addition to the culture medium. Plates were incubated at 37°C in a CO2 incubator for 48 hours. siRNA transfection efficiency was assessed by Western blot and qRT-PCR.

### 2.15. Statistical analysis

GraphPad Prism 8 and IBM SPSS 26.0 were utilized for statistical analyses. Differences between two groups were determined using unpaired two-tailed Student’s t-tests. For comparisons among multiple groups, one-way or two-way analysis of variance (ANOVA) was employed, followed by Tukey’s and Dunnett’s tests, respectively. When analyzing multiple groups with two factors, two-way ANOVA was conducted, followed by Tukey’s multiple comparison test. Specific statistical details are presented in the figure captions and the source data. A p-value of less than 0.05 was considered to indicate statistical significance. Unless otherwise indicated, data from all experiments are displayed as the mean ± standard error of the mean (SEM).

## 3. Results

### 3.1. The abundance of Fusobacterium mortiferum is increased in obese individuals and further enriched in obese adenoma patients

**Figure 1.**
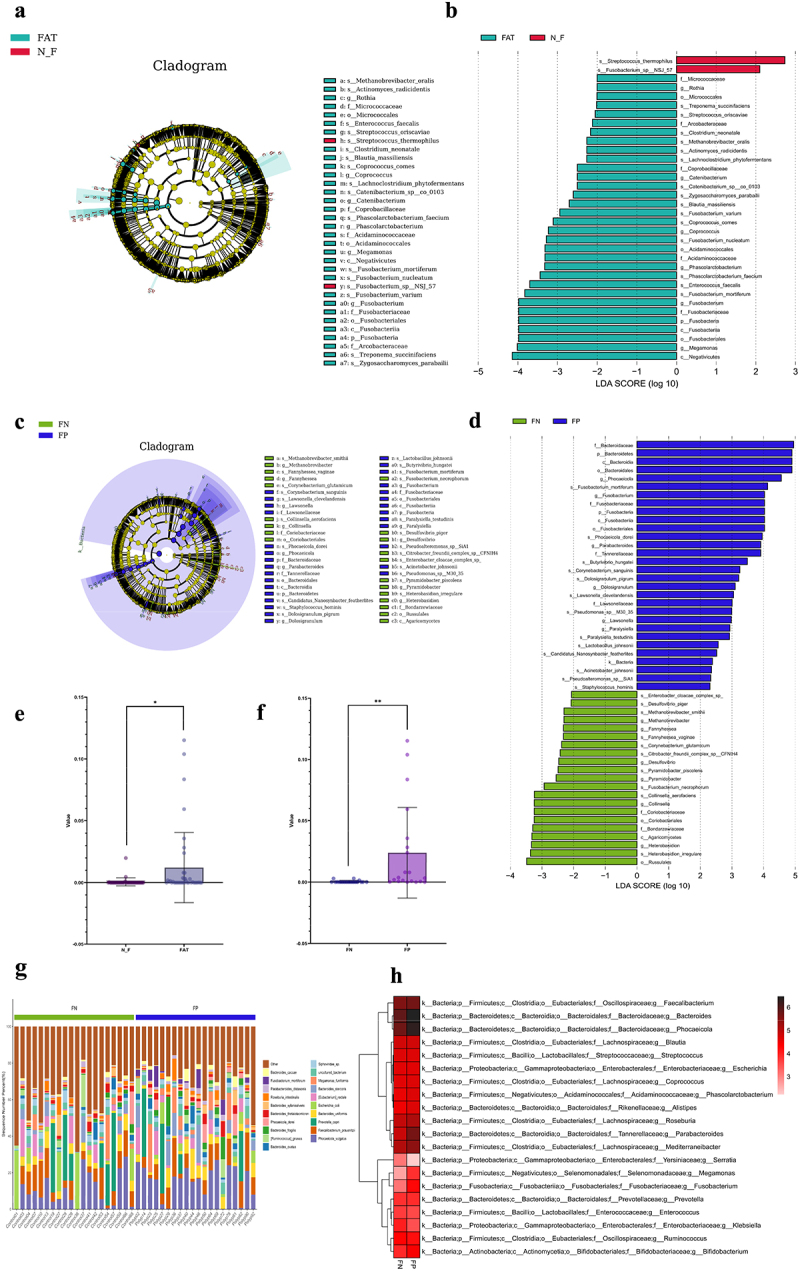
Results of metagenomic analysis and microbiome difference analysis. (a) Preliminary exploration of microbial differences with a Lefse analysis cladogram. Fusobacterium mortiferum is significantly enriched in the obese population (FAT vs. N_F, LDA > 2). (b) An LDA diagram of Lefse analysis (FAT vs. N_F, LDA > 2). (c) In subsequent studies, we further divided the obese population into obese adenoma group (FP) and obese healthy group (FN). A Lefse analysis cladogram of microbial differences shows that Fusobacterium mortiferum is significantly enriched in obese adenoma patients (FP vs. FN, LDA > 2). (d) An LDA diagram of Lefse analysis (FP vs. FN, LDA > 2). (e) Box plot comparing Fusobacterium mortiferum relative abundance across groups (FAT vs. N_F, *p*=0.0159). (f) Box plot comparing Fusobacterium mortiferum relative abundance across groups (FP vs. FN, *p*=0.0092). (g) In the obese population of this study, a bar chart delineates the proportional distribution of samples, with a focus on the 20 species exhibiting the highest relative abundance. (H) A mean heatmap analysis showing the distribution and grouping in FN and FP groups. Error bars ± SEM. *p < 0.05; **p < 0.01; ***p < 0.001; ****p < 0.0001; a single-factor ANOVA coupled with Tukey’s pairwise comparison test was conducted.

We utilized the linear discriminant analysis effect size (LEfSe) analysis with a threshold of LDA ≥ 2 to assess the differences in the abundance of gut microbiota at the species level between obese patients and non-obese individuals (Figure S1). We observed significant enrichment of Phascolarctobacterium faecium, Fusobacterium nucleatum, Fusobacterium varium, Fusobacterium mortiferum, Blautia massiliensis, Zygosaccharomyces parabailii, Catenibacterium sp_co_0103, Lachnoclostridium phytofermentans, Actinomyces radicidentis, Methanobrevibacter oralis, Clostridium neonatale, Streptococcus oriscaviae, and Treponema succinifaciens comes in the gut of obese individuals (Figure 1(a,b)). In addition to Fusobacterium mortiferum, Fusobacterium nucleatum, Fusobacterium varium, and other bacterial species have been reported in literature to be significantly associated with the occurrence of colorectal cancer. To further evaluate the enrichment of gut microbiota in obese patients with adenomatous polyps, we divided the obese cohort into obese adenoma and obese non-adenoma groups. We found that there were differences in bacteria between the obese adenoma group and the obese non-adenoma group, this could be attributed to shifts in the composition of dominant microbial communities and alterations in the host environment. As a result, specific bacterial species, such as Fusobacterium mortiferum, not only demonstrated statistical significance in the initial comparison between obese and non-obese individuals but also exhibited further enrichment in the obese adenoma group (Figure 1(c,d); Table S2). To better illustrate the differences in Fusobacterium mortiferum across groups, we generated a box plot of Fusobacterium mortiferum relative abundance for each group with scatter points to better illustrate the distribution of Fusobacterium mortiferum abundance across groups. The results show that Fusobacterium mortiferum is enriched in obese individuals (Figure 1(e)) and further enriched in obese adenoma patients (Figure 1(f)), with statistically significant differences observed. Simultaneously, we found that the bacterial communities of obese adenoma patients exhibited distinct microbiological characteristics compared to those of the obese non-adenoma group (Figure 1(g)). The mean heatmap analysis of the two groups of people further revealed that there are differences in Fusobacterium mortiferum between the groups (Figure 1(h)). We further validated whether these specific microbial communities also exist differences in non-obese adenoma and healthy populations. Interestingly, the results showed that there was no enrichment of these microbial communities in the non-obese adenoma population (Figure S2). Overall, these data demonstrate that there are significant differences in the distribution and enrichment of the gut microbiota in obese patients with adenomatous polyps, with Fusobacterium mortiferum being a notably differential species.

### 3.2. The untargeted metabolomics of Fusobacterium mortiferum suggested that 5- aminovaleric acid is an important metabolite

**Figure 2.**
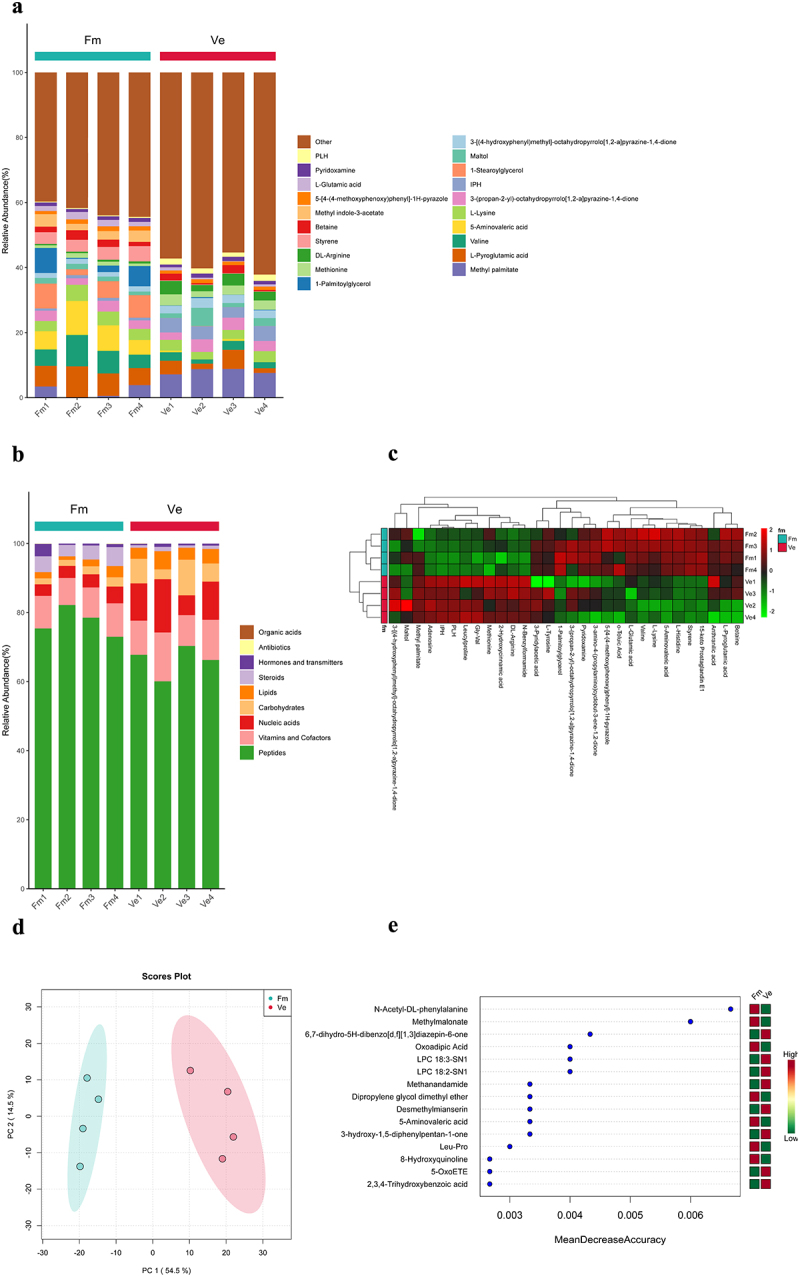
Non-targeted metabolite analysis results of Fusobacterium mortiferum. (a) Stacked bar chart of the top 20 metabolites by percentage content in the two groups. (b) Stacked bar chart of the percentage of metabolites involved in biological processes in the two groups. (c) Heatmap clustering results of metabolites shows 5-aminovaleric acid is significantly enriched in the Fm. group. (d) PCA plot, suggesting a significant difference in the compositional structure of the Fusobacterium mortiferum metabolome compared to the vehicle group. (e) The 15 most important metabolites in the random forest model, indicating significant enrichment of 5-AVA in the metabolome of Fusobacterium mortiferum.

To further explore whether Fusobacterium mortiferum influences the occurrence of adenomas through metabolites, we first conducted an untargeted metabolomics analysis of the metabolites of Fusobacterium mortiferum. We collected the culture medium after the growth and filtration of Fusobacterium mortiferum and performed an untargeted metabolomics analysis with the untreated normal Brain Heart Infusion as the control group. The samples in each group maintained uniformity, making the metabolite analysis more representative. The results of the untargeted metabolomics indicated significant differences in the composition of the metabolome produced by Fusobacterium mortiferum compared to the control group, and 5-AVA appears to be significantly represented among the metabolites of Fusobacterium mortiferum (Figure 2(a)), and the biochemical reactions involved in the two groups are also different (Figure 2(b)). The heatmap shows that, compared to the blank control group, the Fusobacterium mortiferum group is enriched with metabolites such as betaine, L-pyroglutamic acid, styrene, and 5-AVA (Figure 2(c)). Principal component analysis showed clear differences in the metabolic composition between the two groups of samples (Figure 2(d)). We constructed a random forest model with the data and selected the 15 most significantly differentially abundant metabolites, which also demonstrated the enrichment of 5-AVA in the Fusobacterium mortiferum group (Figure 2(e)). Therefore, based on the above results, we have reason to believe that 5-AVA is an important metabolite in Fusobacterium mortiferum.

### 3.3. Fusobacterium mortiferum and 5-AVA enhances the development of intestinal tumors in AOM-mouse model of colorectal cancer

**Figure 3.**
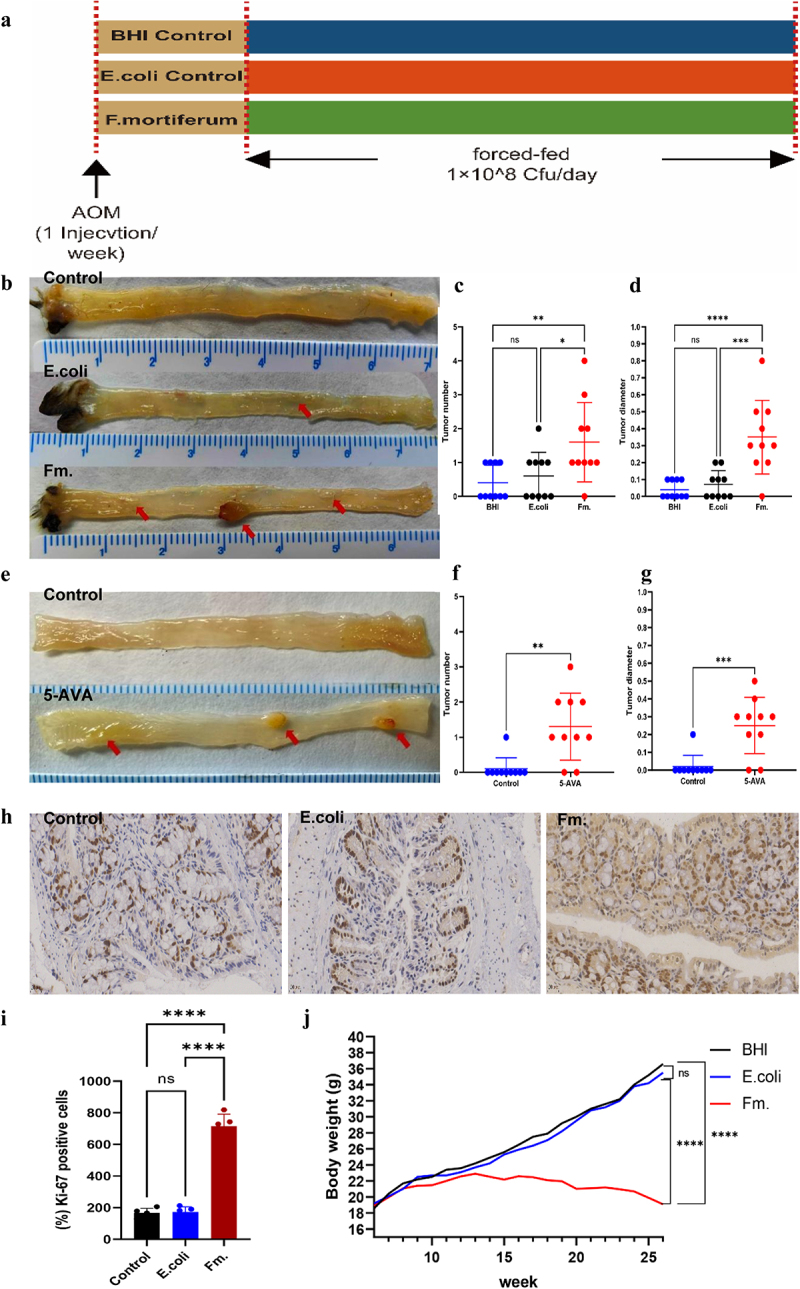
Fusobacterium mortiferum and 5-AVA promotes intestinal tumorigenesis in AOM mice. (a) The experimental design and timing for gavage of microbiota in animal experiments. The mice received daily gavage of either Fusobacterium mortiferum or the non-toxic E. coli at a dosage of 1×10^8^ CFU per mouse, or the culture medium BHI, for a duration of 20 weeks. (b) Rectal and tumor specimens from mice, and tumor marking in the rectum of mice in the Fusobacterium mortiferum (fm.) group and the non-toxic E. coli (E.coli) group. (c) Mice in the Fm. group had a higher number of colorectal tumors compared with the control group (*p*=0.0099) and E. coli group (*p*=0.0343). (d) Mice in the Fm. group had a larger tumor diameter compared with the control group (*p*<0.0001) and E. coli group (*p*<0.0001). (e) Rectal and tumor specimens from mice, and tumor marking in the rectum of mice in the 5-AVA group. The mice received daily gavage of either 5-AVA or PBS at a dosage of 0.2ml per mouse, for a duration of 8 weeks. (f) Mice in the 5-AVA group had a higher number of colorectal tumors compared with the control group (*p*=0.0013). (g) Mice in the 5-AVA group had a higher number of colorectal tumors compared with the control group (*p*=0.005). (h) Representative colonic ki-67 images (scale bar=200 μm) from SPF mice treated by Fusobacterium mortiferum, E. coli, and BHI. (i) Statistical analysis results of ki-67 positive cell count (*p*<0.0001). (j) The body weight values of mice in each group per week after the commencement of gavage (*p*<0.0001). Error bars ± SEM. *p < 0.05; **p < 0.01; ***p < 0.001; ****p < 0.0001; a single-factor ANOVA coupled with Tukey’s pairwise comparison test was conducted. The graphed dots indicate measurements from individual mice; the dual-factor ANOVA was processed with Tukey’s for multiple comparisons (j).

To further assess the tumor-promoting capabilities of Fusobacterium mortiferum and its metabolite 5-AVA in vivo, we colonized antibiotic-treated specific pathogen-free (SPF) mice with Fusobacterium mortiferum, using nontoxic E. coli or the culture medium as controls (Figure 3(a)). As anticipated, mice treated with Fusobacterium mortiferum exhibited accelerated azoxymethane (AOM)-induced colorectal tumor formation, characterized by hematochezia (bloody stools) and rapid weight loss (Figure 3(j)). Moreover, compared to the control and E. coli groups, the growth of colorectal tumors in the Fusobacterium mortiferum group was significantly pronounced (Figure 3(b)). Statistical data on colorectal tumors indicated that, compared to the control and E. coli groups, the number of colorectal tumors (Figure 3(c)) and their maximum diameter (Figure 3(d)) were significantly increased in the Fusobacterium mortiferum group. Subsequently, to further evaluate whether 5-AVA is involved in this tumor promotion process, we administered 5-AVA via gavage for an additional 8 weeks to SPF mice that had undergone the same treatment, with PBS serving as a control. The results depicted in Figure 3(e) show that the growth of AOM-induced colorectal tumors in mice was significantly enhanced following the sustained administration of 5-AVA (Figure 3(e)). Similarly, statistical data on colorectal tumors indicated that, compared to the control group, the number of colorectal tumors (Figure 3(f)) and their maximum diameter (Figure 3(g)) were significantly increased in the 5-AVA gavage group. Immunohistochemical staining of colorectal specimens from mice gavaged with Fusobacterium mortiferum showed a marked increase in the number of Ki-67 positive cells (Figure 3(h)), which was significantly higher than in the control and E. coli groups (Figure 3(i)). The same results were also observed in the 5-AVA group (Figure S3). In summary, our results suggest that Fusobacterium mortiferum and its metabolite 5-AVA promote tumor formation in mice.

### 3.4. Fusobacterium mortiferum and 5-AVA promotes the proliferation of colorectal tumor cells in vitro

**Figure 4.**
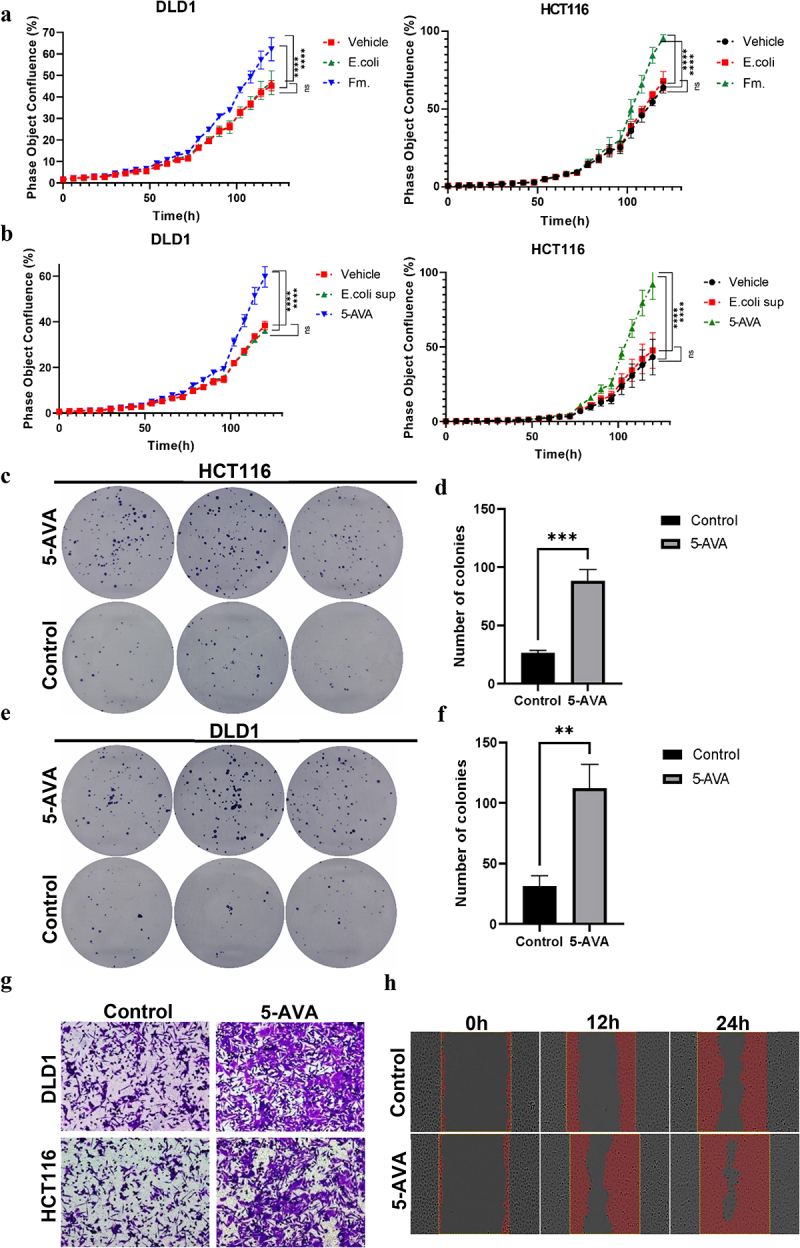
Fusobacterium mortiferum and 5-AVA promote the proliferation of colorectal cancer cells. (a) Co-culture of Fusobacterium mortiferum with colorectal cancer cells (HCT116 and DLD1) promotes cell proliferation (*p*<0.0001). (b) Co-culture of 5-AVA with colorectal cancer cells (HCT116 and DLD1) promotes cell proliferation (*p*<0.0001). (c) Co-culture of 5-AVA with HCT116 cells promotes colony formation. (d) The HCT116 cell clonogenic assay count indicates that the 5-AVA group has a higher number of colony formations compared to the control group (*p*<0.001). (e) Co-culture of 5-AVA with DLD1 cells promotes colony formation. (f) The DLD1 cell clonogenic assay count indicates that the 5-AVA group has a higher number of colony formations compared to the control group (*p*<0.001). (g) The transwell co-culture of DLD1 and HCT116 cell types treated with 5-AVA or PBS, along with representative images of CRC cells migrated upon induction by 5-AVA. The co-cultured cells in the upper and lower compartments of the transwell chamber did not make direct contact. Scale bar, 100 μm. (h) Representative image of a scratch migration assay in HCT116 cells at 0, 12, and 24 h. Error bars ± SEM. *p < 0.05; **p < 0.01; ***p < 0.001; ****p < 0.0001; student t test; two-way ANOVA with Tukey’s multiple comparison test (a, b).

Subsequently, we used the IncuCyte S3 system to assess whether Fusobacterium mortiferum and its metabolite 5-AVA could inhibit the proliferation of colon cancer cells. Under the same treatment conditions, compared with the blank control and negative control groups, Fusobacterium mortiferum significantly enhanced the proliferative vitality of HCT116 and DLD1 cells, and this enhancement was time-dependent (Figure 4(a)). In summary, Fusobacterium mortiferum at a multiplicity of infection (MOI) of 10 enhanced the proliferative vitality of HCT116 and DLD1 cells within 96 hours. We then evaluated the effect of the Fusobacterium mortiferum metabolite 5-AVA on the vitality of the same colon cancer cell lines, and according to previous literature, at a dose of 0.1 mg/L, 5-AVA enhanced the proliferative vitality of HCT116 and DLD1 cells within 96 hours, with the proliferative effect increasing over time (Figure 4(b)). Since Fusobacterium mortiferum at an MOI of 10 and 5-AVA at 0.1 mg/L were sufficient to significantly promote the proliferation of HCT116 and DLD1 cells, the same doses were chosen for subsequent cell stimulation experiments. We also used a colony formation assay to study the proliferative capacity of colon cancer cells. The results showed that compared with the control group, the total number of colonies formed by HCT-116 (Figure 4(c)) and DLD1 (Figure 4(e)) cells co-cultured with 5-AVA increased, and the quantitative results were statistically significant (Figure 4(d,f)). To assess the effect of 5-AVA on tumor invasiveness, we further conducted Transwell and scratch assays. The Transwell (Figure 4(g)) and scratch assay (Figure 4(h)) results indicated that after the same conditions and treatment times, the proliferative and invasive capabilities of colon cancer cells increased, with significant differences in the results. These results suggest that Fusobacterium mortiferum and its metabolite 5-AVA significantly promote the proliferation of colon tumor cells.

### 3.5. Fusobacterium mortiferum and 5-AVA promotes colorectal tumorigenesis by inhibiting DKK2 expression and activating Wnt/β-catenin signaling pathway

**Figure 5.**
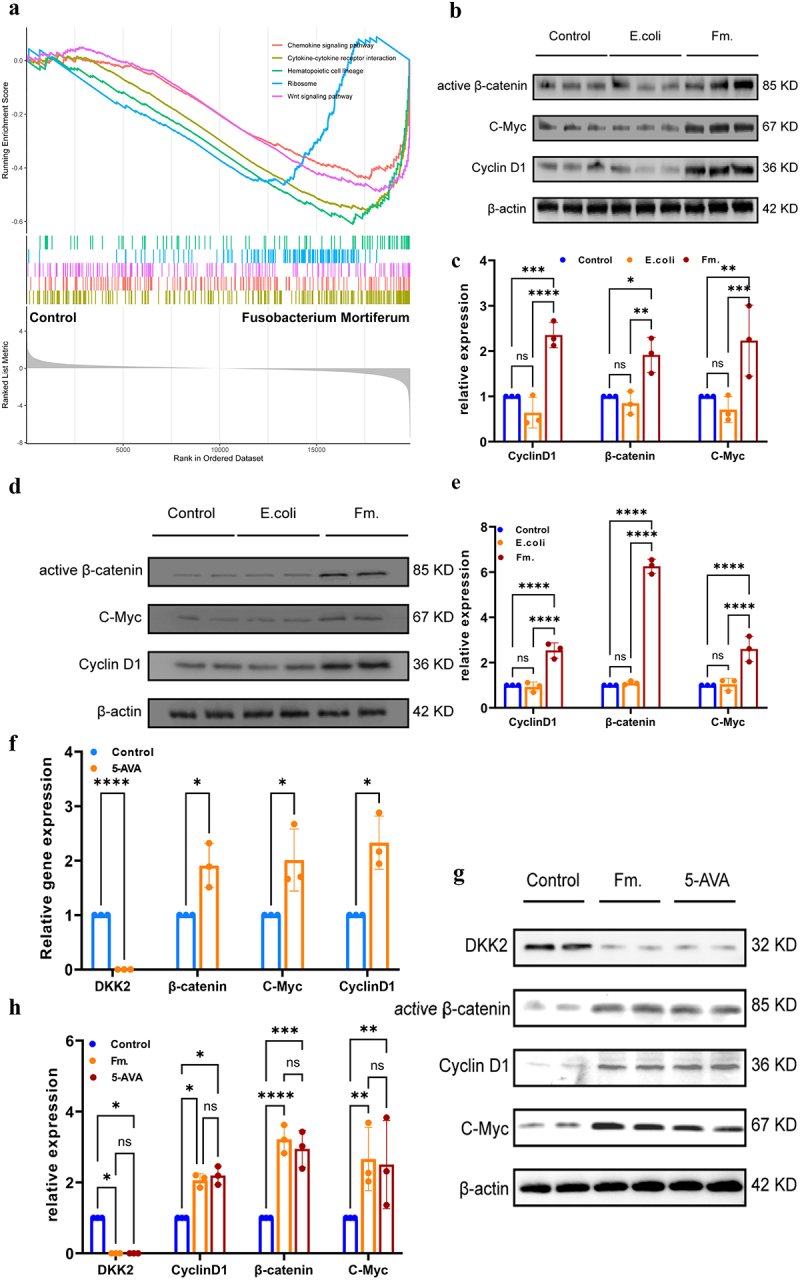
Fusobacterium mortiferum and 5-AVA promotes tumorigenesis through the Wnt/β-catenin pathway. (a) GSEA analysis revealed a significant enrichment in the Wnt/β-catenin signaling pathway in the colorectal tissues of mice treated with Fusobacterium mortiferum by gavage. (*p*=0.0012) (b) Western blot analysis of the mouse colorectal tissues treated with Fm., E.Coli or BHI by gavage. (c) The quantitative data of (b). (d) Western blot analysis of the colorectal cells treated with Fm., E.Coli or PBS. (e) The quantitative data of (d). (f) RT-qPCR analysis from cell experiments suggests that the expression level of DKK2 is decreased in the 5-AVA group, while the expression levels of β-catenin, C-myc, and cyclin D1 are increased. (g) Western blot analysis of the colorectal cells treated with 5-AVA, Fm. Or PBS. (h). The quantitative data of (g) error bars ± SEM. *p < 0.05; **p < 0.01; ***p < 0.001; ****p < 0.0001; a two-way ANOVA with Tukey’s multiple comparison test was conducted; student t test.

To further elucidate the mechanism by which Fusobacterium mortiferum and its metabolite 5-AVA promote tumor growth, we conducted a eukaryotic reference transcriptome analysis on intestinal tissues from mice with the AOM tumor model. The GSEA results (Figure 5(a)) indicated significant differences in the Chemokine signaling pathway, Cytokine-cytokine receptor interaction, Hematopoietic cell lineage, Ribosome, and Wnt signaling pathway in mice treated with Fusobacterium mortiferum compared to the control group. Data analysis and Western blot results from animal tissues (Figure 5(b)) showed a significant increase in the expression of β-catenin and enhanced expression of downstream molecules such as C-myc and Cyclin D1 in mice treated with Fusobacterium mortiferum (Figure 5(c)). Subsequently, we also performed RT-qPCR and Western blot analyses after in vitro bacterial-cell co-culture. The results (Figure 5(d)) showed the same in cells co-cultured with Fusobacterium mortiferum, that is, an increase in the expression of β-catenin, and a significant increase in the expression of key downstream molecules in the Wnt/β-catenin signaling pathway, such as C-myc and Cyclin D1, which were statistically significant (Figure 5(e)). As an important metabolite of Fusobacterium mortiferum, whether 5-AVA can have the same effect in promoting tumor growth is worth further exploration. RT-qPCR analysis from cell experiments suggests the same trend in DKK2 and downstream molecules. (Figure 5(f)). Therefore, we set up the experiment with 5-AVA and cells co-cultured as the experimental group, Fusobacterium mortiferum and cells co-cultured as the positive control group, and PBS and cells co-cultured as the negative control group. The results showed that 5-AVA and Fusobacterium mortiferum similarly inhibit the expression of DKK2 and promote the expression of β-catenin, C-myc, and Cyclin D1 (Figure 5(g)), and are statistically significant (Figure 5(h)). Overall, the results suggest that Fusobacterium mortiferum and its metabolite 5-AVA may inhibit the expression of DKK2 through some mechanism, thereby activating the Wnt/β-catenin pathway and promoting the occurrence of colorectal tumors. Previous literature has reported that DKK2 can act as an adipokine and has an inhibitory effect on the development of obesity, and the suppression of DKK2 expression may further promote the development of obesity, which is consistent with our clinical observation results. This confirms that the Wnt/β-catenin pathway may play an important role in the promotion of tumor proliferation by Fusobacterium mortiferum and its metabolite 5-AVA.

### 3.6. The inhibition of KDM6B-mediated demethylation of DKK2 may play a significant role in the activation of the Wnt/β-catenin signaling pathway by 5-AVA

**Figure 6.**
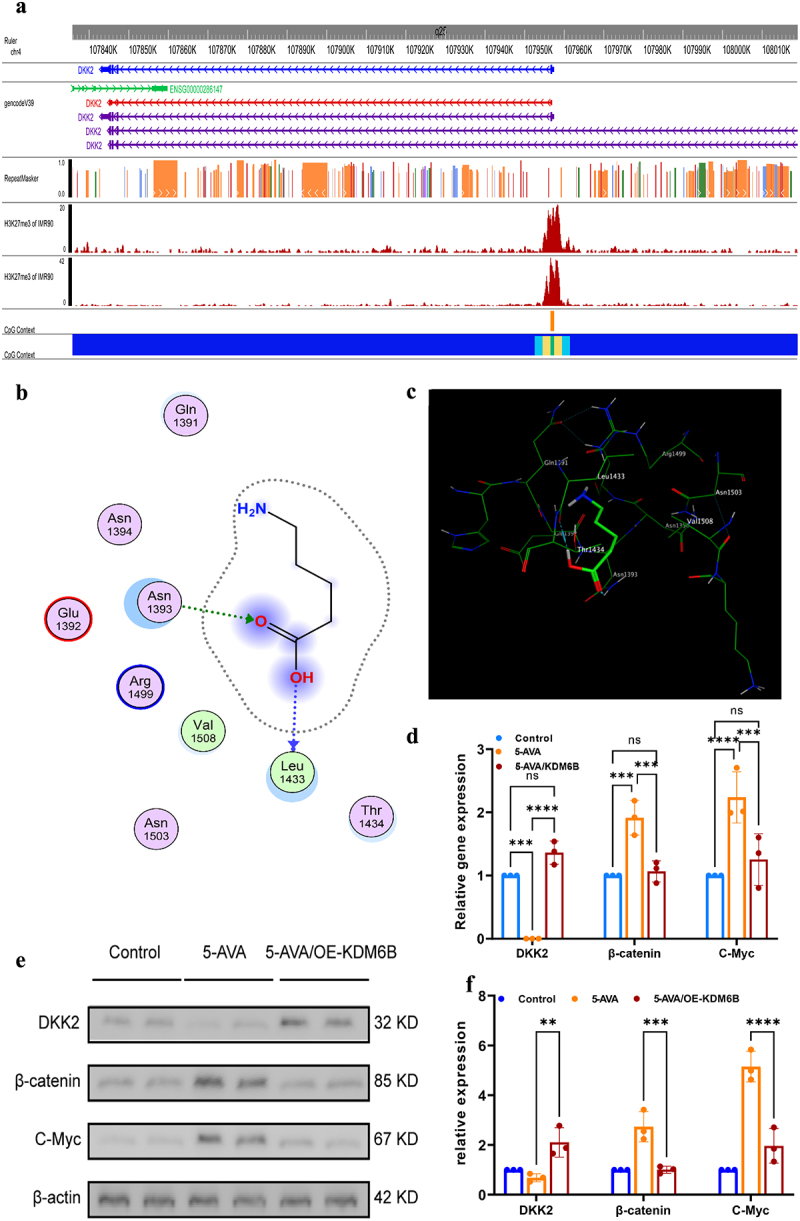
5-AVA may increase DKK2 methylation levels by inhibiting KDM6B. (a) Analysis of DKK2 CpG methylation and H3K27me3 methylation map. (b) Docking of 5-AVA with KDM6B demethylation targets using MOE software, displaying a 2D planar diagram of the interaction between 5-AVA and its target through amino acid side chains and the backbone receptor. (c) 3D simulation diagram of the docking conformation of 5-AVA with KDM6B demethylation targets. (d) RNA-seq analysis from cell experiments suggests that the expression level of DKK2 is increased in the 5-AVA/OE-KDM6B group, while the expression levels of β-catenin, C-myc are decreased. (e) Western blot analysis of the colorectal HCT116 cells treated with 5-AVA, 5-AVA/OE-KDM6B or PBS. (f) The quantitative data of (e). Error bars ± SEM. *p < 0.05; **p < 0.01; ***p < 0.001; ****p < 0.0001; a two-way ANOVA with Tukey’s multiple comparison test was conducted; student t test.

The previous literature has reported that the methylation of DKK2 plays a significant role in the development of colorectal cancer. KDM6B is an important histone demethylase that mediates the demethylation of H3K27me3. We analyzed the methylation levels of DKK2 (Figure 6(a)) and potential H3K27me3 methylation sites online using the genome browser (https://epigenomegateway.wustl.edu/.), and analyzed the potential small molecule-protein binding targets of 5-AVA using the small molecule-protein interaction database (https://prediction.charite.de/.) (Figure S4). The results suggested that KDM6B is a potential binding target for 5-AVA. We then used MOE software for molecular-protein docking validation, and the results confirmed that 5-AVA can bind with KDM6B in various forms (Figures 6(b,c), S5). To confirm whether 5-AVA has an effect on the function of KDM6B, we further conducted in vitro experiments. We set the group with overexpression of KDM6B after adding 5-AVA as the experimental group, the group with only 5-AVA as the positive control group, and the PBS group as the negative control group for comparison. RT-qPCR (Figure 6(d)) and western blot (Figure 6(e)) results showed that the expression of DKK2 increased after overexpression of KDM6B, while the expression of β-catenin, C-myc, and Cyclin D1 decreased, and these changes were statistically significant (Figure 6(f)). In summary, 5-AVA may interact with KDM6B, regulate the Wnt/β-catenin signaling pathway by affecting the methylation of DKK2, and promote the development of colorectal tumors.

## 4. Discussion

The present study aimed to investigate the role of Fusobacterium mortiferum and its metabolite 5-AVA in the development of colorectal cancer and adenoma in obese individuals. Our findings indicate a significant association between the abundance of Fusobacterium mortiferum and the progression of CRC in obese population, suggesting a potential role in the regulation of the Wnt/β-catenin pathway through DKK2 demethylation (Figure S6). In our study, we selected β-catenin, C-myc, and Cyclin D1 as representative biomarkers for the Wnt/β-catenin signaling pathway. These molecules are well-established downstream effectors of the Wnt/β-catenin pathway and their upregulation is directly associated with the progression of colorectal cancer, as supported by previous literature.^23,27^

Our results corroborate previous studies that have implicated the gut microbiome in the development of CRC, particularly in obese individuals. The enrichment of Fusobacterium mortiferum in obese patients with adenomatous polyps highlights its potential as a biomarker for CRC risk in this population. The observed lower bacterial diversity in the obese adenoma group may reflect an altered gut microbiota composition that could contribute to CRC development. To verify Fusobacterium mortiferum’s specificity, we tested representative Fusobacterium nucleatum (Fn.) and Fusobacterium varium (Fv.) strains under the same conditions in vitro and in vivo, and analyzed bacterial culture supernatants via targeted metabolomics. For the in vivo study, we used isogenic nude mice with subcutaneous adenoma organoids to better simulate the actual in vivo environment of adenomas. Over two weeks, mice were gavaged with Fn., Fv., or E. coli. In vitro, these bacteria were co-cultured with colorectal cancer cells and KDM6B-overexpressing colorectal cancer cells to test their effect on KDM6B inhibition and DKK2 expression. Results showed Fusobacterium nucleatum and Fusobacterium varium did not inhibit KDM6B or affect DKK2 expression (Figure S7). Targeted metabolomics revealed significantly higher 5-AVA in Fm. group than in Fn., Fv., and control groups (Table S4). In summary, Fusobacterium mortiferum and its metabolite 5-AVA have species-specific effects on KDM6B and DKK2.

The identification of 5-AVA as a key metabolite in Fusobacterium mortiferum is a novel finding with significant implications. The increase in 5-AVA levels observed in our study is consistent with its potential role in cell necrosis and intestinal inflammation, which are known to be associated with CRC. 5-AVA is a metabolic product of some obligate anaerobes, which can be synthesized by bacteria through the decarboxylation reaction of lysine, and an increase in 5-AVA may indicate cell necrosis.^40^ In mouse experiments, it was observed that 5-AVA is related to intestinal inflammation, it found that the level of 5-AVA increased in the late stage of intestinal inflammation in IL-10 mice due to increased tissue damage caused by inflammation.^41^ In addition, 5-AVA is also involved in the synthesis of heme, and heme is considered an oxidative biological compound, which can increase in a heme-rich diet, thereby increasing the risk of colorectal cancer.^42^ In a serum metabolomics study of breast cancer patients, it was demonstrated that 5-AVA has a predictive role in the occurrence of breast cancer.^43^ In studies related to 5-AVA and colorectal cancer, an increase in the level of 5-AVA is considered to predict the risk of colorectal cancer.^44^

The mechanistic link between Fusobacterium mortiferum, 5-AVA, and Wnt/β-catenin pathway is a critical finding of our study. The downregulation of DKK2, a known inhibitor of the Wnt/β-catenin pathway, suggests that Fusobacterium mortiferum and 5-AVA may promote tumorigenesis by inhibiting DKK2 expression. This is further supported by our in vitro and in vivo experiments demonstrating increased proliferation of CRC cells and activation of the Wnt/β-catenin pathway in the presence of Fusobacterium mortiferum and 5-AVA. The potential interaction between 5-AVA and KDM6B, a histone demethylase, is an intriguing aspect of our study. The inhibition of KDM6B-mediated demethylation of DKK2 by 5-AVA could be a key regulatory mechanism in the activation of the Wnt/β-catenin signaling pathway. This finding warrants further investigation into the epigenetic modifications influenced by the gut microbiome and its metabolites in CRC development.

An interesting point is why Fusobacterium mortiferum is more likely to cause colorectal tumors in obese individuals. As mentioned above, obesity leads to specific changes in the intestinal environment, characterized by chronic low-grade inflammation and compromised intestinal barrier function, which makes it easier for Fusobacterium mortiferum to colonize the intestine. Moreover, the compromised integrity of the intestinal barrier caused by obesity allows Fm. and 5-AVA to more easily penetrate the mucosal barrier and exert pro-tumorigenic effects on colonic epithelial cells. It is worth noting that the inflammatory environment and adipokines related to obesity may affect the expression of DKK2, which is also involved in fat metabolism and the development of obesity.^29^ Therefore, Fm. and 5-AVA further suppress the expression of DKK2, exacerbate obesity-related inflammation, and form a vicious cycle, thereby promoting the occurrence of colorectal tumors. This interaction may explain why Fm. particularly promotes the development of colorectal tumors in obese patients.

While our study provides valuable insights into the role of Fusobacterium mortiferum and 5-AVA in CRC, there are limitations that must be acknowledged. The study’s focus on animal models and in vitro experiments necessitates further validation in human cohorts. The limited number of samples in our study necessitates further validation in multicenter cohorts with larger populations. Additionally, the complex interactions within the gut microbiome and the influence of other microbial metabolites require further exploration. Future research should aim to elucidate the precise molecular mechanisms by which Fusobacterium mortiferum and 5-AVA interact with host cells and the Wnt/β-catenin pathway. Moreover, the development of targeted therapies that modulate these interactions could offer novel treatment strategies for CRC, particularly in obese populations.

**Figure 7.**
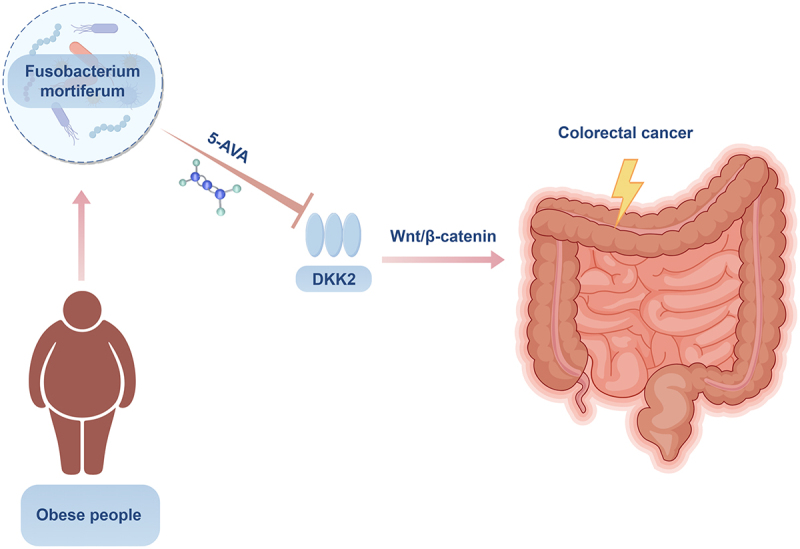
A schematic diagram: Fusobacterium mortiferum and its metabolite 5-AVA as potential promoters of CRC in obese individuals through DKK2 regulation of the Wnt/β-catenin pathway.

In conclusion, our study implicates Fusobacterium mortiferum and its metabolite 5-AVA as potential promoters of CRC in obese individuals through DKK2 regulation of the Wnt/β-catenin pathway (Figure 7). These findings contribute to the growing body of evidence highlighting the importance of the gut microbiome in CRC and provide a foundation for future research and therapeutic development.

## Supplementary Material

Supplemental Material

## Data and materials availability

Sequence data that support the findings of this study have been deposited in the National Center for Biotechnology Information with the primary accession code PRJNA1172275 and MetaboLights database with the primary accession code MTBLS11379.

## References

[1] Sung H, Ferlay J, Siegel RL, Laversanne M, Soerjomataram I, Jemal A, Bray F. Global cancer statistics 2020: GLOBOCAN estimates of incidence and mortality worldwide for 36 cancers in 185 countries. CA A Cancer J Clinicians. 2021 Jan 1. 71(3):209–21. doi: 10.3322/caac.21660. 33538338

[2] Dekker E, Tanis PJ, Vleugels JLA, Kasi PM, Wallace MB. Colorectal cancer. Lancet. 2019 Oct 19. 394(10207):1467–1480. doi: 10.1016/S0140-6736(19)32319-0. PMID: 31631858. 31631858

[3] Wang L, Yu S, Chan ER, Chen KY, Liu C, Che D, Awadallah A, Myers J, Askew D, Huang AY, et al. Notch-regulated dendritic cells restrain inflammation-associated Colorectal carcinogenesis. Cancer Immunol Res. 2021 Mar. 9(3):348–361. doi: 10.1158/2326-6066.CIR-20-0428. 33441309 PMC7925430

[4] Luo M, Wang X, Wu S, Yang C, Su Q, Huang L, Fu K, An S, Xie F, Kkw T, et al. A20 promotes colorectal cancer immune evasion by upregulating STC1 expression to block “eat-me” signal. Signal Transduct Targeted Ther. 2023 Aug 23. 8(1):312. doi: 10.1038/s41392-023-01545-x. PMC10444827 37607946

[5] Wang X, Zhou Y, Ning L, Chen J, Chen H, Li X. Knockdown of ANXA10 induces ferroptosis by inhibiting autophagy-mediated TFRC degradation in colorectal cancer. Cell Death Dis. 2023 Sep 4. 14(9):588. doi: 10.1038/s41419-023-06114-2. 37666806 PMC10477278

[6] Blüher M. Obesity: global epidemiology and pathogenesis. Nat Rev Endocrinol. 2019 May. 15(5):288–298. doi: 10.1038/s41574-019-0176-8. 30814686

[7] Avgerinos KI, Spyrou N, Mantzoros CS, Dalamaga M. Obesity and cancer risk: emerging biological mechanisms and perspectives. Metabolism. 2019 Mar. 92:121–135. doi: 10.1016/j.metabol.2018.11.001. 30445141

[8] Heintzman DR, Fisher EL, Rathmell JC. Microenvironmental influences on T cell immunity in cancer and inflammation. Cell Mol Immunol. 2022 Mar. 19(3):316–326. doi: 10.1038/s41423-021-00833-2. 35039633 PMC8762638

[9] Zhou X, Zhang J, Lv W, Zhao C, Xia Y, Wu Y, Zhang Q. The pleiotropic roles of adipocyte secretome in remodeling breast cancer. J Exp & Clin Cancer Res. 2022 June 14. 41(1):203. doi: 10.1186/s13046-022-02408-z. 35701840 PMC9199207

[10] Kim CE, Yoon L, Michels K, Tranfield W, Jacobs J, May F. The impact of prebiotic, probiotic, and synbiotic supplements and yogurt consumption on the risk of colorectal neoplasia among adults: a systematic review. Nutrients. 2022;14(22):4937. doi: 10.3390/nu14224937. 36432622 PMC9697560

[11] Zhang X, Pan Z. Influence of microbiota on immunity and immunotherapy for gastric and esophageal cancers. Gastroenterol Rep. 2020 June 4. 8(3):206–214. doi: 10.1093/gastro/goaa014. PMC7333930 32665852

[12] Rong J, Liu S, Hu C, Liu C. Single probiotic supplement suppresses colitis‐associated colorectal tumorigenesis by modulating inflammatory development and microbial homeostasis. J Gastroen Hepatol. 2019;34(7):1182–1192. doi: 10.1111/jgh.14516. 30357910

[13] Zou S, Fang L, Lee MH. Dysbiosis of gut microbiota in promoting the development of colorectal cancer. Gastroenterol Rep. 2018 Feb. 6(1):1–12. doi: 10.1093/gastro/gox031. PMC5806407 29479437

[14] Münzker J, Haase N, Till A, Sucher R, Haange SB, Nemetschke L, Gnad T, Jäger E, Chen J, Riede SJ, et al. Functional changes of the gastric bypass microbiota reactivate thermogenic adipose tissue and systemic glucose control via intestinal FXR-TGR5 crosstalk in diet-induced obesity. Microbiome. 2022 June 24. 10(1):96. doi: 10.1186/s40168-022-01264-5. 35739571 PMC9229785

[15] Wang JD, Chen WY, Li JR, Lin SY, Wang YY, Wu CC, Liao SL, Ko CC, Chen CJ. Aspirin mitigated tumor growth in obese mice involving metabolic inhibition. Cells. 2020 Feb 28. 9(3):569. doi: 10.3390/cells9030569. 32121098 PMC7140453

[16] Zhang Y, Zhang L, Zheng S, Li M, Xu C, Jia D, Qi Y, Hou T, Wang L, Wang B, et al. Fusobacterium nucleatum promotes colorectal cancer cells adhesion to endothelial cells and facilitates extravasation and metastasis by inducing ALPK1/NF-κB/ICAM1 axis. Gut Microbes. 2022 Jan. 14(1):2038852. doi: 10.1080/19490976.2022.2038852. 35220887 PMC8890384

[17] Chyan YJ, Poeggeler B, Omar RA, Chain DG, Frangione B, Ghiso J, Pappolla MA. Potent neuroprotective properties against the Alzheimer beta-amyloid by an endogenous melatonin-related indole structure, indole-3-propionic acid. J Biol Chem. 1999 Jul 30. 274(31):21937–21942. doi: 10.1074/jbc.274.31.21937. PMID: 10419516. 10419516

[18] Yagi K, Lange R, Douzou P. Spectroscopic demonstration of an initial stage of the complex of D-amino acid oxidase and its substrate D-alpha-aminobutyric acid. Biochem Biophys Res Commun. 1980 Nov 28. 97(2):370–374. doi: 10.1016/0006-291x(80)90274-0. PMID: 6110424. 6110424

[19] Martínez Y, Li X, Liu G, Bin P, Yan W, Más D, Valdivié M, Hu CA, Ren W, Yin Y. The role of methionine on metabolism, oxidative stress, and diseases. Amino Acids. 2017 Dec. 49(12):2091–2098. doi: 10.1007/s00726-017-2494-2. Epub 2017 Sep 19. PMID: 28929442. 28929442

[20] Dikici S, Mangır N, Claeyssens F, Yar M, MacNeil S. Exploration of 2-deoxy-D-ribose and 17β-estradiol as alternatives to exogenous VEGF to promote angiogenesis in tissue-engineered constructs. Regen Med. 2019 Mar. 14(3):179–197. doi: 10.2217/rme-2018-0068. Epub 2019 Feb 22. PMID: 30793662. 30793662

[21] Liang S, Mao Y, Liao M, Xu Y, Chen Y, Huang X, Wei C, Wu C, Wang Q, Pan X, et al. Gut microbiome associated with APC gene mutation in patients with intestinal adenomatous polyps. Int J Biol Sci. 2020 Jan 1. 16(1):135–146. doi: 10.7150/ijbs.37399. 31892851 PMC6930378

[22] Kim S, Song G, Lee T, Kim M, Kim J, Kwon H, Kim J, Jeong W, Lee U, Na C, et al. Parsylated transcription factor EB (TFEB) regulates the expression of a subset of wnt target genes by forming a complex with β-catenin-TCF/LEF1. Cell Death Differ. 2021 Sep. 28(9):2555–2570. doi: 10.1038/s41418-021-00770-7. 33753903 PMC8408140

[23] Deng F, Zhou R, Lin C, Yang S, Wang H, Li W, Zheng K, Lin W, Li X, Yao X, et al. Tumor-secreted dickkopf2 accelerates aerobic glycolysis and promotes angiogenesis in colorectal cancer. Theranostics. 2019 Jan 30. 9(4):1001–1014. doi: 10.7150/thno.30056. 30867812 PMC6401398

[24] Hirata H, Hinoda Y, Nakajima K, Kawamoto K, Kikuno N, Kawakami K, Yamamura S, Ueno K, Majid S, Saini S, et al. Wnt antagonist gene DKK2 is epigenetically silenced and inhibits renal cancer progression through apoptotic and cell cycle pathways. Clin Cancer Res. 2009 Sep 15. 15(18):5678–5687. doi: 10.1158/1078-0432.CCR-09-0558. PMID: 19755393. 19755393

[25] Shen T, Chen Z, Qiao J, Sun X, Xiao Q. Neutralizing monoclonal antibody against Dickkopf2 impairs lung cancer progression via activating NK cells. Cell Death Discov. 2019 Jul 31. 5:123. doi: 10.1038/s41420-019-0204-4. 31372243 PMC6668384

[26] Chumduri C, Gurumurthy RK, Berger H, Dietrich O, Kumar N, Koster S, Brinkmann V, Hoffmann K, Drabkina M, Arampatzi P, et al. Opposing wnt signals regulate cervical squamocolumnar homeostasis and emergence of metaplasia. Nat Cell Biol. 2021 Feb. 23(2):184–197. doi: 10.1038/s41556-020-00619-0. 33462395 PMC7878191

[27] Farkas SA, Vymetalkova V, Vodickova L, Vodicka P, Nilsson TK. DNA methylation changes in genes frequently mutated in sporadic colorectal cancer and in the DNA repair and Wnt/β-catenin signaling pathway genes. Epigenomics. 2014 Apr. 6(2):179–191. doi: 10.2217/epi.14.7. PMID: 24811787. 24811787

[28] Zhou Q, Wan Q, Jiang Y, Liu J, Qiang L, Sun L. A landscape of murine long non-coding RNAs reveals the leading transcriptome alterations in adipose tissue during aging. Cell Rep. 2020 May 26. 31(8):107694. doi: 10.1016/j.celrep.2020.107694. 32460027 PMC7603645

[29] Yang J, Shi BY. Dickkopf (dkk)-2 is a beige fat-enriched adipokine to regulate adipogenesis. Biochem Biophys Res Commun. 2021 Apr 9. 548:211–216. doi: 10.1016/j.bbrc.2021.02.068. 33647798

[30] Bradley D, Deng T, Shantaram D, Hsueh WA. Orchestration of the adipose tissue immune landscape by adipocytes. Annu Rev Physiol. 2024 Feb 12. 86(1):199–223. doi: 10.1146/annurev-physiol-042222-024353. 38345903

[31] Gomes AC, Hoffmann C, Mota JF. The human gut microbiota: metabolism and perspective in obesity. Gut Microbes. 2018 Jul 4. 9(4):308–325. doi: 10.1080/19490976.2018.1465157. Epub 2018 May 24. PMID: 29667480; PMCID: PMC6219651. 29667480 PMC6219651

[32] Sánchez-Alcoholado L, Ordóñez R, Otero A, Plaza-Andrade I, Laborda-Illanes A, Medina JA, Ramos-Molina B, Gómez-Millán J, Queipo-Ortuño MI. Gut microbiota-mediated inflammation and Gut permeability in patients with obesity and colorectal cancer. Int J Mol Sci. 2020 Sep 16. 21(18):6782. doi: 10.3390/ijms21186782. 32947866 PMC7555154

[33] Jiang Y, Li F, Gao B, Ma M, Chen M, Wu Y, Zhang W, Sun Y, Liu S, Shen H. KDM6B-mediated histone demethylation of LDHA promotes lung metastasis of osteosarcoma. Theranostics. 2021 Feb 6. 11(8):3868–3881. doi: 10.7150/thno.53347. 33664867 PMC7914357

[34] Qiao Y, Li L, Bai L, Gao Y, Yang Y, Wang L, Wang X, Liang Z, Xu JT. Upregulation of lysine-specific demethylase 6B aggravates inflammatory pain through H3K27me3 demethylation-dependent production of TNF-α in the dorsal root ganglia and spinal dorsal horn in rats. CNS Neurosci Ther. 2023 Nov. 29(11):3479–3492. 37287407 10.1111/cns.14281 PMC10580362

[35] Liu F, Wang Y, Yang Z, Cui X, Zheng L, Fu Y, Shao W, Zhang L, Yang Q, Jia J. KDM6B promotes gastric carcinogenesis and metastasis via upregulation of CXCR4 expression. Cell Death Dis. 2022 Dec 23. 13(12):1068. doi: 10.1038/s41419-022-05458-5. 36564369 PMC9789124

[36] Zhang J, Ying Y, Li M, Wang M, Huang X, Jia M, Zeng J, Ma C, Zhang Y, Li C, et al. Targeted inhibition of KDM6 histone demethylases eradicates tumor-initiating cells via enhancer reprogramming in colorectal cancer. Theranostics. 2020 Aug 8. 10(22):10016–10030. doi: 10.7150/thno.47081. 32929331 PMC7481431

[37] D’Oto A, Fang J, Jin H, Xu B, Singh S, Mullasseril A, Jones V, Abu-Zaid A, von Buttlar X, Cooke B, et al. KDM6B promotes activation of the oncogenic CDK4/6-pRB-E2F pathway by maintaining enhancer activity in MYCN-amplified neuroblastoma. Nat Commun. 2021 Dec 10. 12(1):7204. doi: 10.1038/s41467-021-27502-2. 34893606 PMC8664842

[38] Consultation, W. H. O. Obesity: preventing and managing the global epidemic. Report of a WHO consultation. World Health Organ Tech Rep Ser. 2000;894:1–253. PMID: 11234459. 11234459

[39] WHO Expert Consultation. Appropriate body-mass index for Asian populations and its implications for policy and intervention strategies. Lancet. 2004 Jan 10. 363(9403):157–163. doi: 10.1016/S0140-6736(03)15268-3. 14726171

[40] Hmdb.ca. Human metabolome database: showing metabocard for 5-aminopentanoic acid (HMDB0003355). 2020 [accessed on 2020 Nov 30]. https://Hmdb.ca/Metabolites/HMDB0003355.

[41] Lin HM, Barnett MP, Roy NC, Joyce NI, Zhu S, Armstrong K, Helsby NA, Ferguson LR, Rowan DD. Metabolomic analysis identifies inflammatory and noninflammatory metabolic effects of genetic modification in a mouse model of Crohn’s disease. J Proteome Res. 2010 Apr 5. 9(4):1965–1975. doi: 10.1021/pr901130s. PMID: 20141220. 20141220

[42] Martin OCB, Olier M, Ellero-Simatos S, Naud N, Dupuy J, Huc L, Taché S, Graillot V, Levêque M, Bézirard V, et al. Haem iron reshapes colonic luminal environment: impact on mucosal homeostasis and microbiome through aldehyde formation. Microbiome. 2019 May 6. 7(1):72. doi: 10.1186/s40168-019-0685-7. PMID: 31060614; PMCID: PMC6503375. 31060614 PMC6503375

[43] Lécuyer L, Dalle C, Lyan B, Demidem A, Rossary A, Vasson MP, Petera M, Lagree M, Ferreira T, Centeno D, et al. Plasma metabolomic signatures associated with long-term breast cancer risk in the SU.VI.MAX prospective cohort. Cancer Epidemiol, Biomarker & Prev. 2019 Aug. 28(8):1300–1307. doi: 10.1158/1055-9965.EPI-19-0154. Epub 2019 Jun 4. PMID: 31164347. 31164347

[44] Bisht V, Nash K, Xu Y, Agarwal P, Bosch S, Gkoutos GV, Acharjee A. Integration of the microbiome, metabolome and Transcriptomics Data identified novel metabolic pathway regulation in colorectal cancer. Int J Mol Sci. 2021 May 28. 22(11):5763. doi: 10.3390/ijms22115763. PMID: 34071236; PMCID: PMC8198673. 34071236 PMC8198673

