# Supplementary material for: Fusobacterium mortiferum and its metabolite 5-aminovaleric acid promote the development of colorectal cancer in obese individuals through Wnt/β-catenin pathway by DKK2
Source: Gut Microbes. 2025 May 8;17(1):2502138. doi: 10.1080/19490976.2025.2502138 (PMC12064068; doi:10.1080/19490976.2025.2502138)
Supplement: Supplemental Material [file KGMI_A_2502138_SM5513.zip › supplementary_Wnt_catenin_pathway_by_DKK2.docx]

Supplementary Materials for

**Fusobacterium mortiferum and its metabolite 5-Aminovaleric acid promote the development of colorectal cancer in obese individuals through Wnt/β-catenin pathway by DKK2**

Jiaxin Deng *et al.*

*Corresponding author:

Xuefeng Guo. Email: guoxfeng@mail.sysu.edu.cn

DeZheng Lin. Email: lindzh8@mail.sysu.edu.cn

**This PDF file includes:**

Supplementary Text

Figs. S1 to S7#

Tables S1 to S4#

**Supplementary Text**

**Experimental Procedures**

**Mouse Body Weight Measurement**

In this research, we measured mouse body weight using a standardized method. Mice were kept in consistent environmental conditions and weighed weekly with a calibrated scale. Post-measurement, weights were recorded and compared to previous data. General health was monitored, and data were systematically analyzed for statistical weight changes. This ensured accurate and reliable weight measurements for our study.

**Measurement and collection of colotectal tumors and tissue**

Intestinal tissues were harvested and sectioned into three equal parts, categorized as proximal, medial, and distal segments. Tumors within the intestine were categorized based on their diameter into three distinct size groups: small (less than 1 mm), medium (between 1 and 2 mm), and large (greater than 2 mm). The intestinal Swiss roll preparations were subsequently immersed in a 10% neutral-buffered formalin solution for fixation. Concurrently, adenomatous lesions of the intestinal tissues were cryopreserved at a temperature of -80°C to ensure long-term preservation.

**Ki-67 immunohistochemical staining**

Colonic tissue sections, 2 micrometers in thickness, underwent deparaffinization and were subjected to blocking procedures. They were subsequently incubated with the primary anti-Ki-67 antibody at a dilution of 1:500 at 4 degrees Celsius for a duration of 24 hours. The immunoreaction was visualized using the IHC Select Immunoperoxidase Secondary Detection System, following the protocol provided by the manufacturer. For nuclear counterstaining, Haematoxylin was applied. A negative control was established by utilizing rabbit serum at an equivalent concentration to that of the primary antibody. Microscopic imaging was performed with a light microscope integrated with the Metafer Automatic Slide Scanning and Imaging System. The quantification of Ki-67 positive cells within randomly selected fields was executed using the Image J software.

**DNA Extraction, Gene Sequencing, and Identification**

DNA was extracted from microbial community analysis samples using the bead-beating method with a DNeasy Blood and Tissue Kit from QIAGEN. The hypervariable V4 region of the 16S rRNA gene was amplified via PCR with dual barcode primers as previously described. Subsequently, the denatured amplicon mixture was combined with 20% PhiX Control v.3 and sequenced on the Illumina Hiseq 2500 platform, yielding 2×250 bp paired-end reads. The resulting sequences were processed using Quantitative Insights into Microbial Ecology 2 (QIIME II) software with reference parameters for demultiplexing and quality filtering. The obtained sequences were analyzed, compared, and identified through blast.ncbi.nlm.nih.gov.

**GSEA (gene-set enrichment analysis)**

Gene set enrichment analysis is conducted using the GSEA module of Genepattern (<https://genepattern.broadinstitute.org/>). Our RNA-seq dataset was analyzed by GSEA with gene sets from the Kyoto Encyclopedia of Genes and Genomes (KEGG) pathways. The genetic profiles of all WNT pathway genes, including somatic mutations and copy number variations, were obtained from the CCLE data repository (<https://portals.broadinstitute.org/ccle/>).

**MOE dock**

The active site of the receptor was constructed using the MOE-Site Finder tool, followed by the utilization of MOE-Dock for the positioning of ligands into this site. For the positioning technique, the Triangle Matcher algorithm was engaged, with the London scoring function employed to evaluate the interactions, and the process was parameterized to retain 10 poses for each ligand. Subsequent analysis of the receptor-ligand complexes was conducted to identify the binding interactions and to ascertain the most favorable docking conformation. The selection of the most accurately docked complex, presumed to accurately reflect the interactions between protein and ligand, was guided by the docking score, the similarity of the ligand's orientation within the active site to that of the reference ligands, and the preservation of key interactions. The efficacy of this docking methodology was confirmed through the successful recovery of the co-crystal ligand's pose when it was docked into its respective binding site within the crystal structure. All visual representations of the complexes were generated using the MOE software, version 2016.0802.

**5-AVA targeted metabolic analysis**

Take 50μL of standard curve working solution into a 1.5mL polypropylene centrifuge tube, add 200μL of 80% methanol-water, vortex to mix, take the supernatant into a vial for LC-MS/MS analysis. Take 50μL of sample into a 1.5mL polypropylene centrifuge tube, add 200μL of 80% methanol-water, vortex mix for 5 minutes, centrifuge (4℃, 15000rpm) for 5 minutes, take the supernatant into a vial for LC-MS/MS analysis.

**Colorectal Adenoma Organoid Culture Method**

Colorectal adenoma samples obtained from surgical specimens were quickly sent to the laboratory in tissue preservation solution at 4°C. The samples were rinsed repeatedly (at least 20 times) with D-PBS pre-cooled at 4°C and containing 5 µM Y-27632 and 1% double antibiotics. The adenoma samples were then placed on a pre-cooled ice box at 4°C and cut into 1-3 mm³ pieces using surgical scissors. The tissues were collected into a 15 mL centrifuge tube and digested with 5 mL of TrypLE™ Express (12605-010, Gibco). The tissue suspension was shaken every 5 min until no large tissue chunks were visible, resulting in small cell clusters. The cell suspension was then filtered through a 100 µm cell strainer and centrifuged at 300g for 3 min. The cell suspension was mixed with Matrigel (VM002-PRF-10, VIVO Matter) at a 1:2 ratio, and the cells were resuspended. The mixture was seeded into a 12-well plate at 75 µL per well. The plate was inverted and incubated in a 37°C, 5% CO₂ cell culture incubator for 30 min. After incubation, 1 mL of colorectal adenoma organoid culture medium was added to each well, and the medium was changed every 3 days. When most organoids exceeded 100 µm in diameter, they were passaged. The medium in the well plate was removed, and 1 mL ofp TryLE™ Express (12605-010, Gibco) was added to digest the organoids for 3 min until the cell clusters were smaller than 15 µm. The digestion was stopped, and colore thectal adenoma organoids were reseeded into the culture plate as described above.

**Colorectal Adenoma Organoid Xenotransplantation**

Colorectal adenoma organoids were dissociated into single cells by treatment with TrypLE™ Express (12605-010, Gibco) at 37°C for 3 min, and the digestion was stopped by adding three volumes of D-PBS containing 5 µM Y-27632. The cells were centrifuged at 300g for 3 min, and then mixed with Matrigel (VM002-PRF-10, VIVO Matter) to prepare a cell suspension at a concentration of 1×10^7 cells/mL. Each 6-week-old BALB/c-nu mouse was subcutaneously injected with 100 µL of the cell suspension (1×10^6 cells per mouse) in the right axilla. The mice were randomly divided into four groups (Fm., Fv., Fn., and control group). The Fm. group was gavaged with 200 µL of Fm. bacterial suspension (1×10^9 CFU/mL), the Fv. group with 200 µL of Fv. bacterial suspension (1×10^9 CFU/mL), the Fn. group with 200 µL of Fn. bacterial suspension (1×10^9 CFU/mL), and the control group with 200 µL of PBS. Gavage was performed twice a week. Tumor growth was monitored twice a week with calipers until the mice were euthanized three weeks after inoculation. Tumor volume (V, in mm³) was calculated using the modified ellipsoid formula: V = (L × W²)/2, where L is the length and W is the width.

**Fig. S1.**

**Comparative analysis of gut microbiota composition between obese and non-obese individuals.** Analysis of microbiota composition (A), Venn diagrams (B), and heatmap (C) showing the differential distribution of microbiota in obese and non-obese populations.

**Fig. S2.**

**A comparative analysis of gut microbiota composition between adenoma patients and healthy individuals in the non-obese population.** Analysis of microbiota composition (A), LEfSe cladogram (B), LEfSe LDA diagram (C), and heatmap (D), showing the differential distribution of microbiota between adenoma patients and healthy individuals in the non-obese population The results show that Fusobacterium mortiferum is not included in the differences.


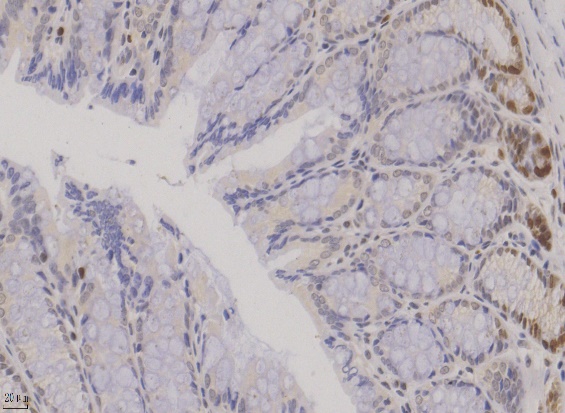

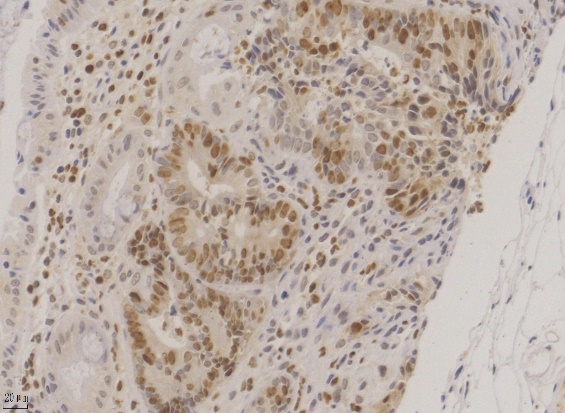


A

B


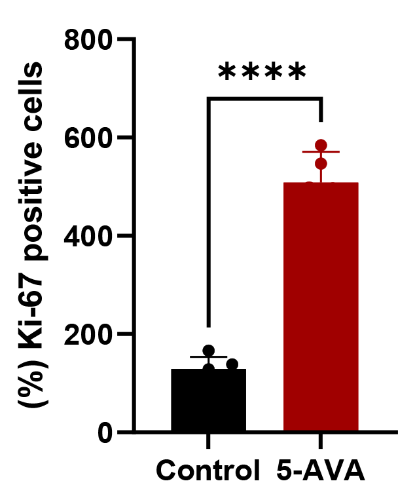


C


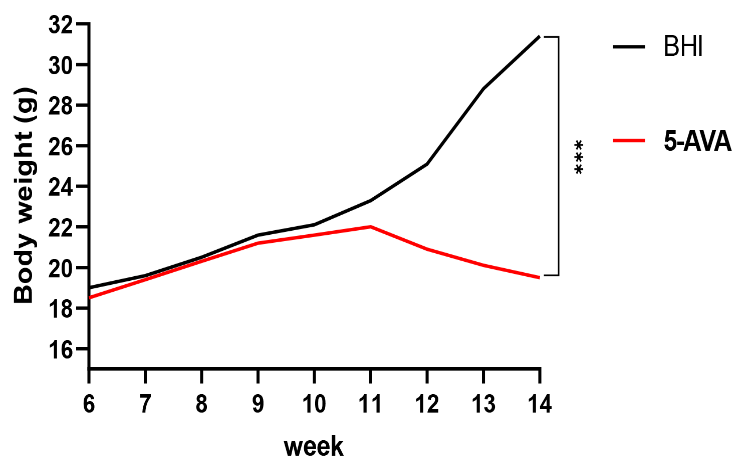


**Control**

**5-AVA**

**Fig. S3.**

**The effect of 5-AVA gavage on mouse growth.** (A) Ki-67 staining of mouse colorectal tissue (B) Quantitative analysis of Ki-67 in the 5-AVA group and control group (C) Weight records of mice after 8 weeks of gavage.


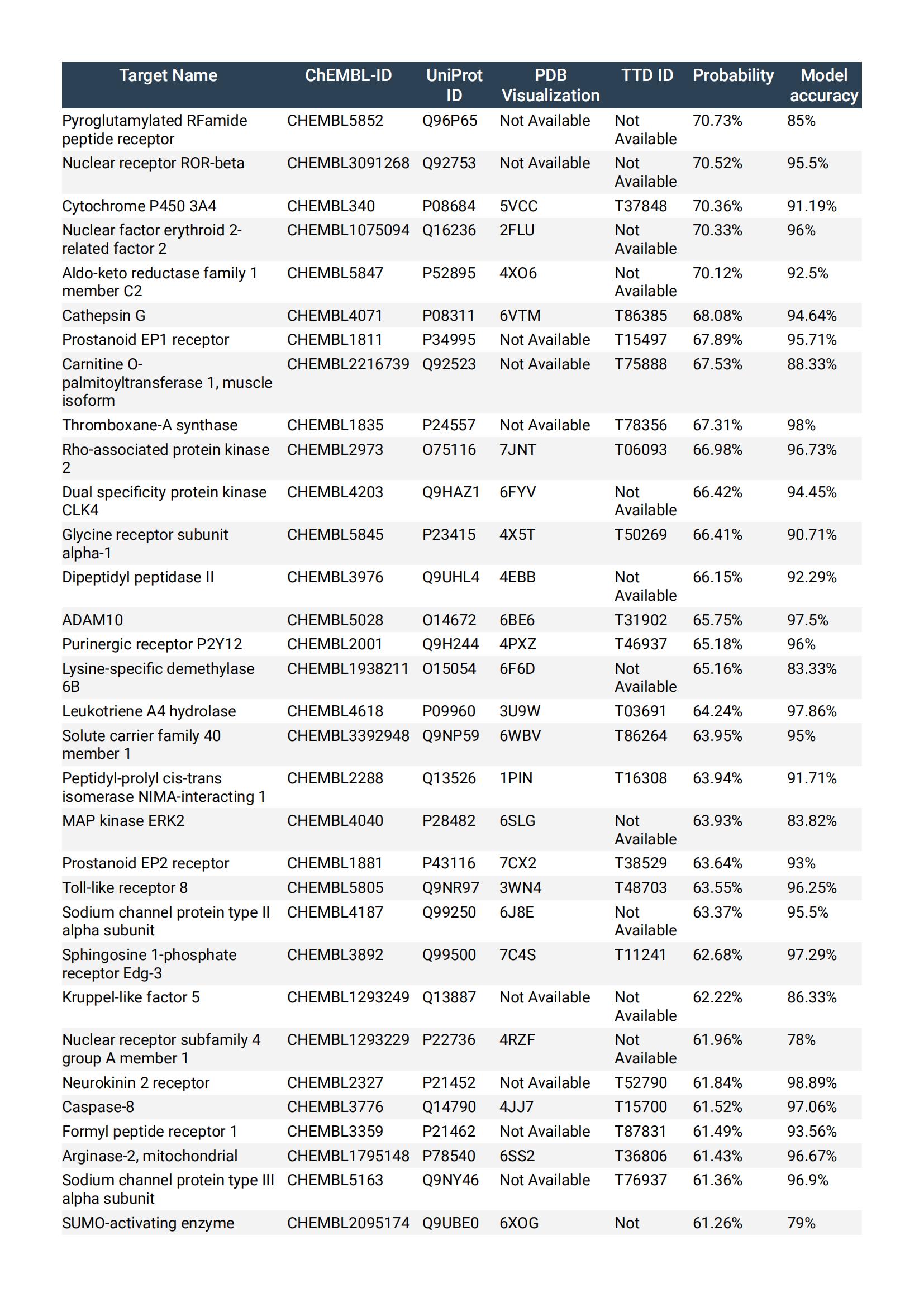


**Fig. S4.**

**the potential small molecule-protein binding targets of 5-AVA using the small molecule-protein interaction database** (<https://prediction.charite.de/>)


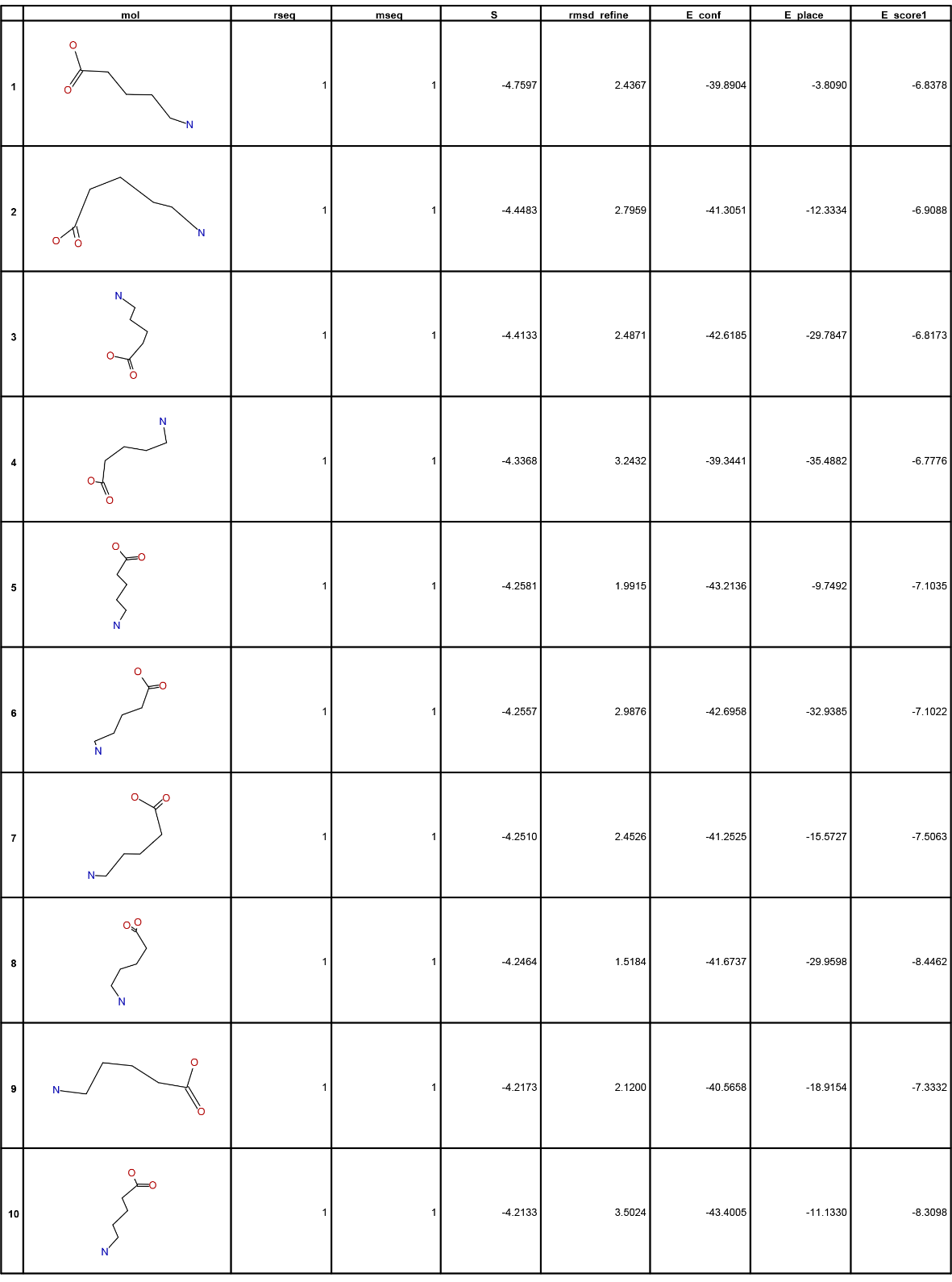


**Fig. S5.**

**MOE molecular simulation of the 10 most likely conformations of 5-AVA binding to KDM6B and their binding scores.**


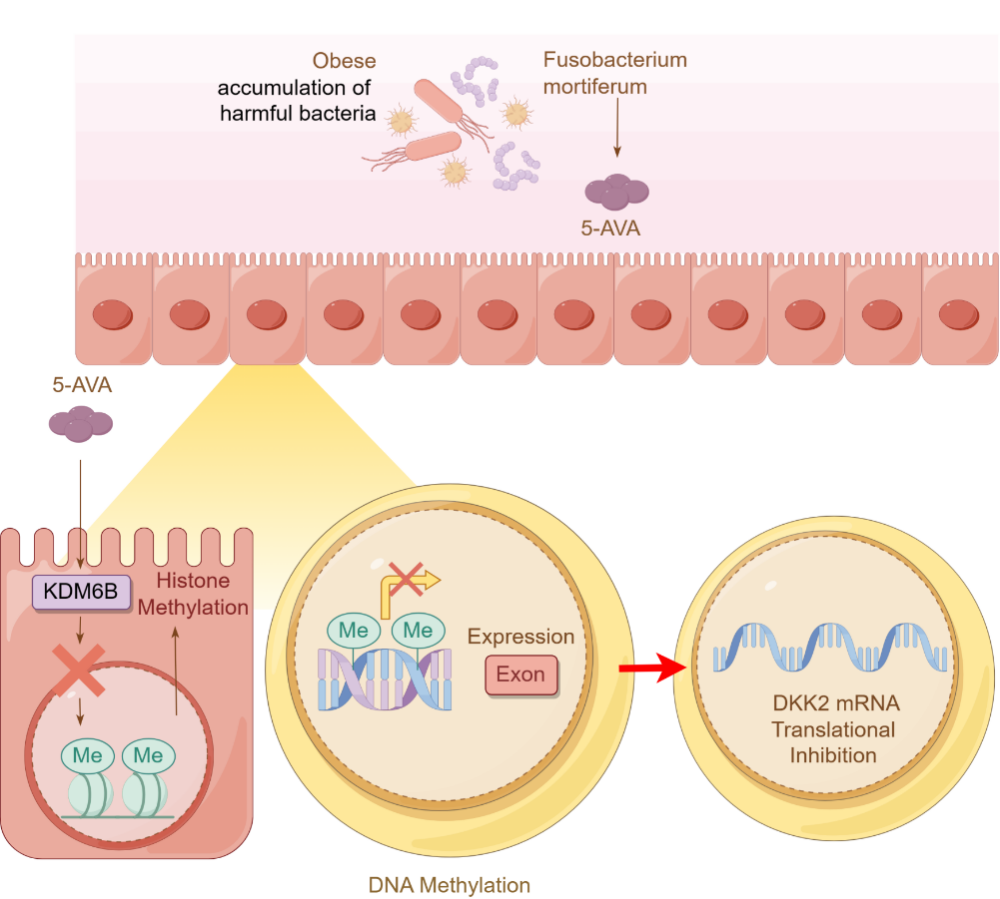


**Fig. S6**

**Summary schema illustrating the thought map of how Fusobacterium mortiferum and 5-AVA promote tumorigenesis in obese populations.**


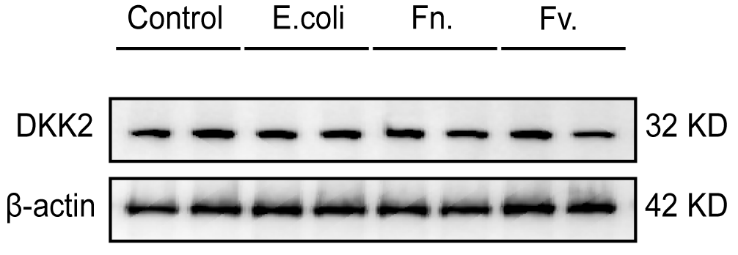

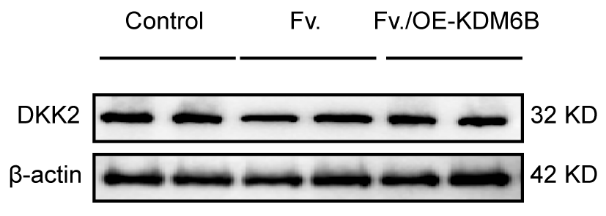

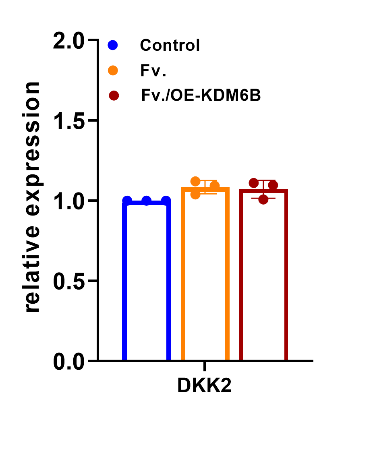

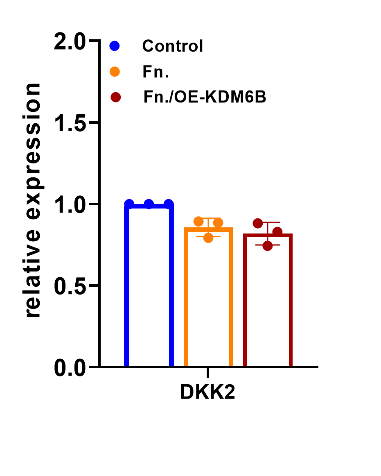

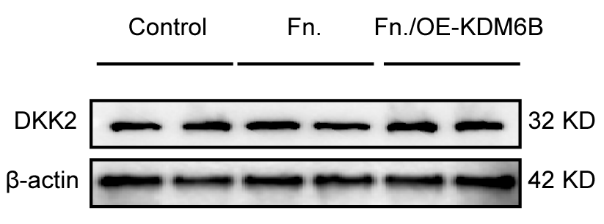

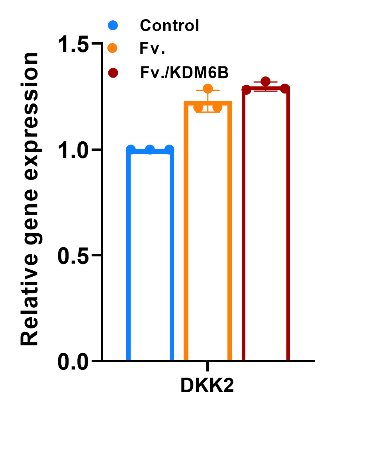

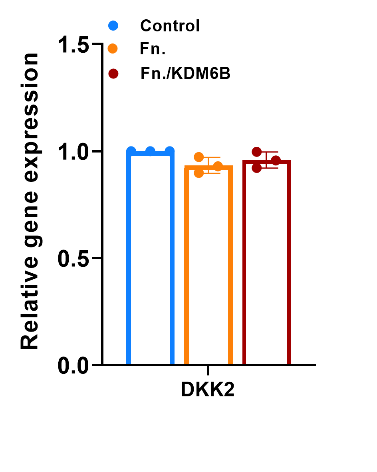

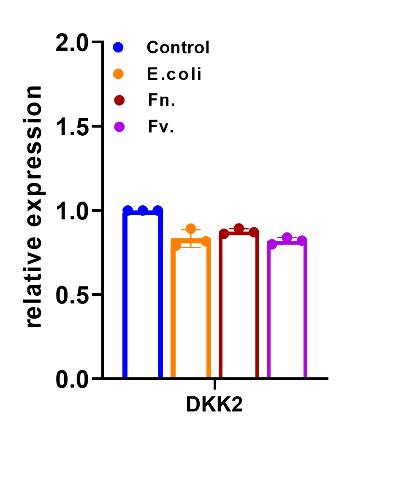

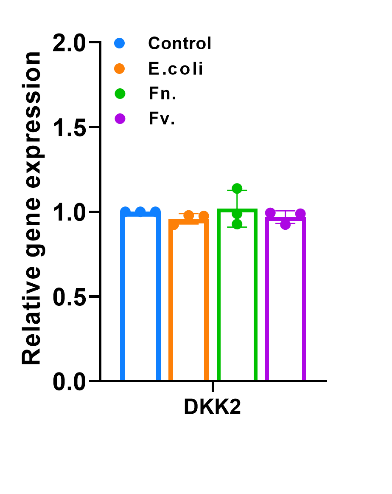


B

H

I

G

F

E

D

A

C

**Fig. S7**

**Fusobacterium nucleatum and Fusobacterium varium exert no significant effects on KDM6B and DKK2 in vitro and in vivo. (A)** RNA-seq analysis from animal experiments indicates that the expression level of DKK2 shows no significant difference among the Fn., Fv., E.coli or BHI by gavage. **(B)** Western blot analysis of the mouse colorectal tissues treated with Fn., Fv., E.coli or BHI by gavage. **(C)** The quantitative data of (B). **(D)** RNA-seq analysis from cell experiments indicates that Fn. exert no significant effects on KDM6B and DKK2 in vitro. **(E)** RNA-seq analysis from cell experiments indicates that Fv. exert no significant effects on KDM6B and DKK2 in vitro.

**(F)** Western blot analysis of the colorectal HCT116 cells treated with Fn., Fn./KDM6B or PBS. **(G)** The quantitative data of (F). **(H)**. Western blot analysis of the colorectal HCT116 cells treated with Fv., Fv./KDM6B or PBS. **(I**). The quantitative data of (H). Error bars ± SEM. *p < 0.05; **p < 0.01; ***p < 0.001; ****p < 0.0001; a two-way ANOVA with Tukey’s multiple comparison test was conducted; student t test.

**Table S1. Clinical information of obese individuals with (FP) and without (FN) adenomas involved in this study**

| **FP individuals (n=20)** | | | **FN individuals (n=20)** |
| --- | --- | --- | --- |
| Gender | Male | 13 | 7 |
|  | Female | 7 | 13 |
| Median BMI | | 25.68 | 25.90 |
| Median age (year) | | 54.75 | 51.9 |
| Diabetes Yes | | 1 | 2 |
| No | | 19 | 18 |
| Hypertension Yes | | 7 | 6 |
| No | | 13 | 14 |
| Smoking Yes | | 6 | 3 |
| No | | 14 | 17 |
| Alcohol Consumption Yes | | 9 | 8 |
| No | | 11 | 12 |
| regular diet | meat-based diet | 6 4 | |
|  | vegetable-based diet  well-balanced diet | 3 3  11 13 | |
|  |  |  | |

**Table S2 The strains identified from obese population with and without adenoma: microbial communities identified by LEfSe analysis (LDA>2, p<0.05)**

| **Strains identified from obese population**  **with adenoma** | **Family/Genus** |
| --- | --- |
| *Fusobacterium_mortiferum* | *Fusobacteriaceae* |
| *Phocaeicola_dorei* | *Flavobacteriaceae* |
| *Butyrivibrio_hungatei* | *Lachnospiraceae* |
| *Corynebacterium_sanguinis* | *Corynebacteriaceae* |
| *Dolosigranulum_pigrum* | *Streptococcaceae* |
| *Lawsonella_clevelandensis* | *Lawsonellaceae* |
| *Pseudomonas_sp__M30_35* | *Pseudomonadaceae* |
| *Paralysiella_testudinis* | *Paralysiella* |
| *Lactobacillus_johnsonii* | *Lactobacillaceae* |
| *Candidatus_Nanosynbacter_featherlites* | *Nanosynbacter* |
| *Acinetobacter_johnsonii* | *Moraxellaceae* |
| *Pseudoalteromonas_sp__SiA1* | *Pseudoalteromonadaceae* |
| *Staphylococcus_hominis* | *Staphylococcaceae* |
| **Strains identified from obese population** |  |
| **without adenoma** | **Family** |
| *Heterobasidion_irregulare* | *Meripilaceae* |
| *Collinsella_aerofaciens*  Fusobacterium_necrophorum  Pyramidobacter_piscolens  Citrobacter_freundii_complex_sp__CFNIH4  Corynebacterium_glutamicum  Fannyhessea_vaginae  Methanobrevibacter_smithii  Desulfovibrio_piger  Enterobacter_cloacae_complex_sp_ | *Coriobacteriaceae*  *Fusobacteriaceae*  *Prevotellaceae*  *Enterobacteriaceae*  *Corynebacteriaceae*  *Fannyhessea*  *Methanobacteriaceae*  *Desulfovibrionaceae*  *Enterobacteriaceae* |

**Table S3. Primer sequences used in this study.**

| **Primers** | **Forward** | **Reverse** |
| --- | --- | --- |
| **C-myc** | CACTAACATCCCACGCTCTGA | AAATCATCGCAGGCGGAACA |
| **CyclinD1** | GAGGCGGAGGAGAACAAACA | GGAGGGCGGATTGGAAATGA |
| **DKK2** | CTCTGGATGGTACTCGGCAC | ATGATCGTAGGCAGGGGTCT |
| **β-catenin** | AGGAATGAAGGTGTGGCGACA | TGGCAGCCCATCAACTGGAT |

| Sample Name | Calculated Concentration (nM) |
| --- | --- |
| Fm.-1  Fm.-2  Fm.-3 | \| 294 \| \| --- \| \| 323 \| \| 298 \| |
| Fn.-1  Fn.-2  Fn.-3 | No Peak  No Peak  No Peak |
| Fv.-1  Fv.-2  Fv.-3 | No Peak  No Peak  No Peak |
| Control-1 | 5.53 |
| Control-2 | 2.21 |
| Control-3 | No Peak |
|  |  |
|  |  |

|  |  |
| --- | --- |

**Table S4. 5-AVA targeted metabolic analysis.**
