# Supplementary material for: Fusobacterium mortiferum and its metabolite 5-aminovaleric acid promote the development of colorectal cancer in obese individuals through Wnt/β-catenin pathway by DKK2
Source: Gut Microbes. 2025 May 8;17(1):2502138. doi: 10.1080/19490976.2025.2502138 (PMC12064068; doi:10.1080/19490976.2025.2502138)
Supplement: Supplemental Material [file KGMI_A_2502138_SM5513.zip › 600 dpi Figure.zip!/Figure2.docx]

**E**

**D**

**C**

**B**

**A**
